# Single-cell analysis of dup15q syndrome reveals developmental and postnatal molecular changes in autism

**DOI:** 10.1038/s41467-025-61184-4

**Published:** 2025-07-04

**Authors:** Yonatan Perez, Dmitry Velmeshev, Li Wang, Matthew L. White, Clara Siebert, Jennifer Baltazar, Guolong Zuo, Juan Andrés Moriano, Songcang Chen, David M. Steffen, Natalia Garcia Dutton, Shaohui Wang, Brittney Wick, Maximilian Haeussler, Stormy Chamberlain, Arturo Alvarez-Buylla, Arnold Kriegstein

**Affiliations:** 1 Eli and Edythe Broad Center of Regeneration Medicine and Stem Cell Research, University of California, San Francisco, San Francisco, CA USA; 2 Department of Neurology, University of California, San Francisco, San Francisco, CA USA; 3 Genomics Institute, University of California, Santa Cruz, CA USA; 4 Departments of Genetics and Genome Sciences and Pediatrics, Connecticut Children’s Medical Center, University of Connecticut Health Center, 400 Farmington Avenue, Farmington, CT USA; 5 Present Address: Bryan Research Building, Duke University, Durham, NC USA

**Keywords:** Autism spectrum disorders, Autism spectrum disorders, Disease model

## Abstract

Duplication 15q (dup15q) syndrome is a leading genetic cause of autism spectrum disorder, offering a key model for studying autism-related mechanisms. Using single-cell and single-nucleus RNA sequencing of cortical organoids from dup15q patient-derived iPSCs and post-mortem brain samples, we identify increased glycolysis, disrupted layer-specific marker expression, and aberrant morphology in deep-layer neurons during fetal-stage organoid development. In adolescent-adult postmortem brains, upper-layer neurons exhibit heightened transcriptional burden related to synaptic signaling, a pattern shared with idiopathic autism. Using spatial transcriptomics, we confirm these cell-type-specific disruptions in brain tissue. By gene co-expression network analysis, we reveal disease-associated modules that are well preserved between postmortem and organoid samples, suggesting metabolic dysregulation that may lead to altered neuron projection, synaptic dysfunction, and neuron hyperexcitability in dup15q syndrome.

## Abstract (web summary)

Single-cell analysis of patient brains and cortical organoids links disrupted developmental metabolic reprogramming to postnatal synaptic dysfunction in dup15q syndrome, revealing shared neuronal changes with idiopathic autism.

## Introduction

Autism spectrum disorder (ASD) is a complex neurodevelopmental disorder with a strong genetic contribution^1^. Despite the heterogeneity of clinical manifestations and underlying genetics, studies of autism patient brains suggest convergence of disease pathology on common pathways^1–11^. Although many studies indicate that some ASD-associated gene modules are co-expressed during prenatal development (early to mid-fetal), other studies highlight ASD-associated genes expressed during postnatal development^2–8^. Recently, organoid models have enabled identification of the cell-type-specific pathology of monogenic and idiopathic ASD^9–11^, and have the potential to further advance our understanding of early developmental phenotypes associated with ASD. Postnatally, specific brain regions underlying higher-order cognitive processes, such as the prefrontal and temporal cortex and the limbic system, have been demonstrated to be specifically affected in ASD^5,7,12–14^. In addition to idiopathic cases, ASD is also associated with several genetic syndromes. The most common is 15q11-q13 duplication syndrome (dup15q syndrome), accounting for up to 3% of all ASD cases^15^. Dup15q is commonly caused by a de-novo maternally derived duplication of the Prader-Willi/Angelman critical region (PWACR), typically encompassing tens of genes (depending on the DNA breaking point). The duplication mainly occurs in two forms: an extra isodicentric 15 chromosome idic(15q) or an interstitial duplication int.dup(15q). Most dup15q patients meet the criteria for ASD. This study aims to gain insight into the cell-type-specific developmental and postnatal molecular changes of dup15q syndrome and explore potential dysregulated programs that converge between dup15q and idiopathic ASD. In this study, we use single-nucleus RNA sequencing (snRNAseq) of dup15q postmortem samples from three different cortical regions, as well as single-cell RNA sequencing (scRNAseq) of iPSC-derived cortical organoids, to model dup15q-associated transcriptional changes throughout development and in adulthood. Our findings provide new insights into how early molecular disruptions in dup15q syndrome might contribute to the pathophysiology observed in the adult brain.

## Results

### Comprehensive single-cell molecular profiling of dup15q

**Fig. 1:**
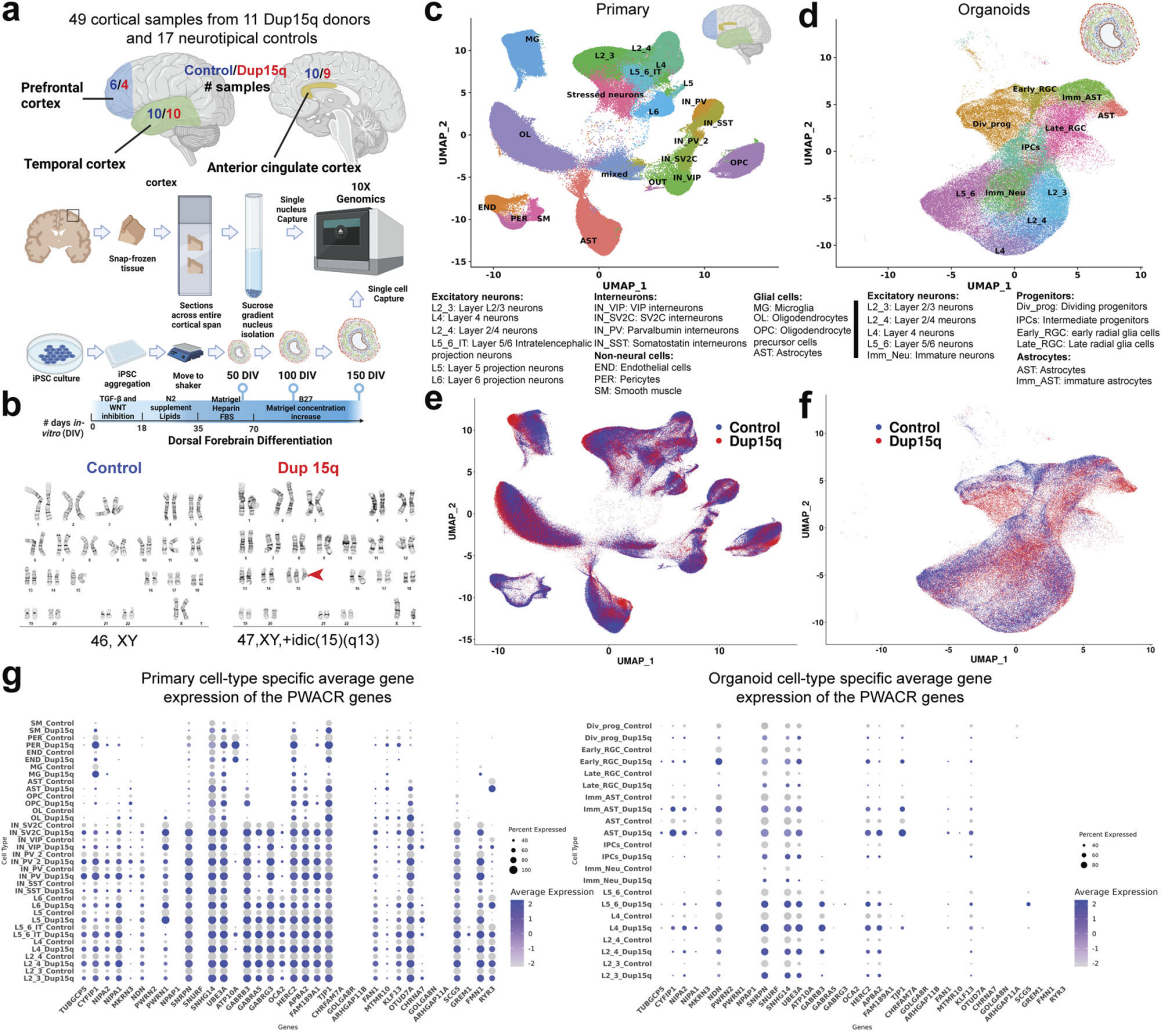
Comprehensive single-cell molecular profiling of dup15q syndrome using postmortem cortical samples and cortical organoids. **a** Illustration of experimental design, sample collection, and cell capture. Some elements were created in BioRender. Perez, J. (2025) https://BioRender.com/nvvwyh3. **b** G-banding karyotype of normal and idic(15q) iPSC lines. **c** Unbiased clustering of single nuclei and annotated cell types of postmortem samples. Some elements were created in BioRender. Perez, J. (2025) https://BioRender.com/6z5et7u. **d** Unbiased clustering of cortical organoid single cells and annotated cell types. Some elements were created in BioRender. Perez, J. (2025) https://BioRender.com/rpjlezb. **e** Primary nuclei clustered by genotype, showing equal contribution of dup15q and control samples to all cell types. **f** Organoid cells clustered by genotype, showing similar contributions from dup15q and control organoids to all cell types. **g** Cell-type-specific average expression of the duplicated genes within the PWACR region in primary and organoid cells.

We performed nuclei isolation and snRNA-seq of 49 snap-frozen post-mortem tissue samples (referred to below as primary tissue) from the prefrontal (PFC), temporal (TC), and anterior cingulate (ACC) cortical regions of 11 dup15q patients and 17 neurotypical controls (Fig. 1a). All dup15q patients were diagnosed with ASD. All samples were matched for age, sex, RNA integrity number (RIN), and post-mortem interval (Supplementary Fig. S1a–e; Supplementary Dataset S1). Tissue was lysed, and nuclei were extracted by ultracentrifugation using a sucrose gradient. Nuclei capture and library preparation were done using the 10x Genomics platform. In total, we generated 345,861 single-nuclei gene expression profiles that passed our quality control standards (methods); 145,346 from dup15q subjects and 200,515 from neurotypical controls (Fig. 1a). For cortical organoids, we used three patient-derived dup15q and three control human induced pluripotent (hiPSC) stem cell lines (Supplementary Fig. S1b, Supplementary Dataset S1). To confirm the duplication of 15q and the integrity of control lines, all lines underwent karyotype analysis before cortical organoid differentiation (Supplementary Fig. S1f). Cortical organoids were generated using a dorsal forebrain differentiation protocol^16,17^, and cultures from different lines demonstrated expected and comparable differentiation progression throughout sampled time points and across genotypes (Supplementary Fig. S1g). Single cells were captured at 50, 100, and 150 days of in vitro differentiation (DIV) using the 10x genomics platform. We generated 106,302 expression profiles from organoid cells: 63,500 from dup15q and 42,802 from controls across three in vitro developmental timepoints (Fig. 1a).

We detected a median of 3108 genes and 7103 transcripts per primary nuclei and a median of 2672 genes and 7908 transcripts per organoid cell. In both datasets, we observed glutamatergic neurons expressing higher numbers of genes and transcripts compared to other cell types (Supplementary Fig. S2a, b). Dimensionality reduction and unbiased clustering of postmortem nuclei from all cortical regions and organoid cell profiles were done separately. Clusters were annotated based on the expression of cell type markers (Supplementary Fig. S2c, d) for molecularly defined cell types annotated in the Allen Brain Atlas^18^. After removing a cluster of neuronal debris contaminated by ambient RNA^19^ (Methods, Supplementary Fig. S2e-g), we identified 17 specific cell types from primary nuclei (ten neuronal, four glial, and three vascular-related cell types) and 11 from cortical organoids (Fig. 1c, d). Primary cell clusters largely agreed with a reference annotation of the human motor cortex (Azimuth; Supplementary Fig. S2h). Organoid clustering identified two radial glia populations (early RGCs and late RGCs), consistent with ventricular radial glia identity^20^ (Supplementary Fig. S2g). We also found dividing and intermediate progenitors and known subtypes of excitatory neurons with identity, matching specific cortical layers (Fig. 1d, Supplementary Fig. S2c). In addition, we found two clusters of GABAergic interneurons. These were subdivided into early GABAergic neurons (mainly arising from organoids at 50 DIV) with a lateral ganglionic eminence (LGE)-like identity and a late interneuron identity (more prominent in organoids at 100 and 150 DIV) with a putative caudal ganglionic eminence (CGE)-like identity^21,22^ (Supplementary Fig. S3a). However, these clusters were present in only a subset of the lines and were thus removed from further analysis (Supplementary Fig. S3b). Nuclei were captured from different samples, and all cortical regions were well intermixed, contributing to all cell types. The cell contribution from different organoids was also uniform, indicating a lack of strong batch effect (Fig. 1c–e, Supplementary Fig. S3c). Organoid cells clustered by days of in vitro differentiation demonstrated sequential emergence and differentiation of excitatory neurons and astrocytes (Supplementary Fig. S1g, Supplementary Fig. S3d-e). The fraction of dup15q and control nuclei contributing to each cell type was relatively uniform across cell types (Supplementary Fig. S3f). As expected, cell contribution was more heterogeneous and variable between organoids from different lines (Supplementary Fig. S3g), likely due to the influence of individual line-specific genomic contexts^10^. To explore the relative cellular abundance between dup15q and controls, we used DCATS for cell proportion analysis^23^. We detected significant changes in cell proportions in the brains of dup15q patients (increase in UL neurons and PV interneurons) but not in organoids (Supplementary Dataset S2). We have more power to detect changes in brain tissue than in organoids due to higher sample size, but it is also possible that these differences could stem from sampling variability.

### Vulnerability of dup15q excitatory neurons throughout development

To identify cell-type specific differential gene expression of dup15q in organoids and primary tissue we used MAST^24^, a framework that performs a zero-inflated regression analysis by fitting a linear mixed model (LMM) to exclude gene expression changes that might stem from confounders. To further reduce variability arising from potentially different maturation rates and differentiation ability in individual organoids, an equal number of cells contributing to each cell type was selected from each sample (Methods). In primary tissue, we identified 7896 differential expression events (q-value < 0.05; expression fold change ≥ 14%) in 3634 unique genes across all cell types. Of these, 4809 genes were upregulated (~61%), and 3087 were downregulated (~39%) (Supplementary Dataset S3). In Organoids, we identified 2914 differentially expressed genes (DEGs) in 1657 unique genes across cell types. Of these, 1341 were upregulated (~46%) and 1573 were downregulated (~54%) (Supplementary Dataset S4). We used pseudobulk analysis to compare our cell type-specific DEGs in primary tissue with bulk dup15q DEGs from the most recent and comprehensive dataset^8^. We found over 600 DEGs, ~42% of which were shared with the bulk dataset with a very high gene expression correlation (Pearson’s r = 0.93, *P*-value = 5.31E-114). However, within cell type-specific DEGs identified in primary tissue by snRNA-seq, only 2868 ( ~ 36%) were also identified by the previous bulk analysis^8^. Therefore, our analysis identified 5028 cell type-specific DEGs that were not detected by bulk analysis (Supplementary Dataset S5, Supplementary Fig. S4a). We used RNA in situ hybridization to validate some of the most differentially expressed genes across all cell types in brain tissue sections (Supplementary Fig. S4b). When we compared gene expression fold changes between organoid and corresponding primary excitatory neurons, we found a significant yet low correlation (i.e., the union of all genes expressed in either primary or organoid cells) (Supplementary Fig. S4c). Genes within the 15q11.2-q13.1 duplicated locus (such as *UBE3A*, *GABRB5*, *GABARG3*, *NIPA2*, and *HERC2*) were among the highest and most recurrently overexpressed genes across all cell types in both organoids and primary tissue (Supplementary Fig. S5a, Fig. 1g, Supplementary Datasets S3-4). Consistent with previous observations^15^, we found 21 duplicated genes upregulated in primary tissue and 13 upregulated in organoids (Supplementary Fig. S5b). Thus, a majority of the differentially expressed genes are located outside the duplicated region, indicating that most dup15q gene dysregulation is not driven by DNA dose-dependent expression alone. Several duplicated genes were most differentially expressed specifically in excitatory neurons (Fig. 1g), suggesting cell type-specific mechanisms that can modulate the expression of genes in the 15q11.2-q13.1 locus. Despite potential organoid line heterogeneity, we found similar cell type-specific average gene expression trends of genes within the PWACR region between all organoid lines (Supplementary Fig. S5c). As expected, we found significant discordance in overall gene expression levels between primary and organoid cell types (Supplementary Fig. S5d). Notably, within PWACR genes, we found some differences, including relatively lower overall gene overexpression, but also groups of genes that showed little or no difference in the organoid model (e.g., *OTUD7A, SCG5, FMN1*, and *RYR3*). This is likely due to the different developmental stages of organoids compared to postmortem tissue. Additionally, we found that within primary cells, overexpression of some PWACR genes was more profound in neurons than in other cell types, consistent with previous observations^25^. This is likely due to either low non-neuronal specific gene expression or due to cell type-specific maternal imprinting. Importantly, there was relatively good agreement of cell-specific PWACR gene expression changes between organoid and primary cells, specifically in excitatory neurons (Fig. 1g). Transcriptome-wide, we found statistically significant DEG overlap between organoids and primary tissue. We found that DL neurons had the most overlapping DEGs between organoids and postmortem tissue (45 genes, accounting for >10% of all organoid DEGs and 7% of primary corresponding cells; *P* = 0.042, Fisher’s exact test) while having the lowest proportion of overlapping duplicated genes (only ~11% of overlapping DEGs were duplicated genes). Aside from L2_3, excitatory neurons showed a significant statistical DEG overlap between primary tissue cells and organoid cells. Excitatory neurons, aside from L4, also showed a significant correlation between expression fold changes of overlapping DEGs (Pearson’s *P*-value < 0.05; Supplementary Fig. S5e, Supplementary Dataset S4).

**Fig. 2:**
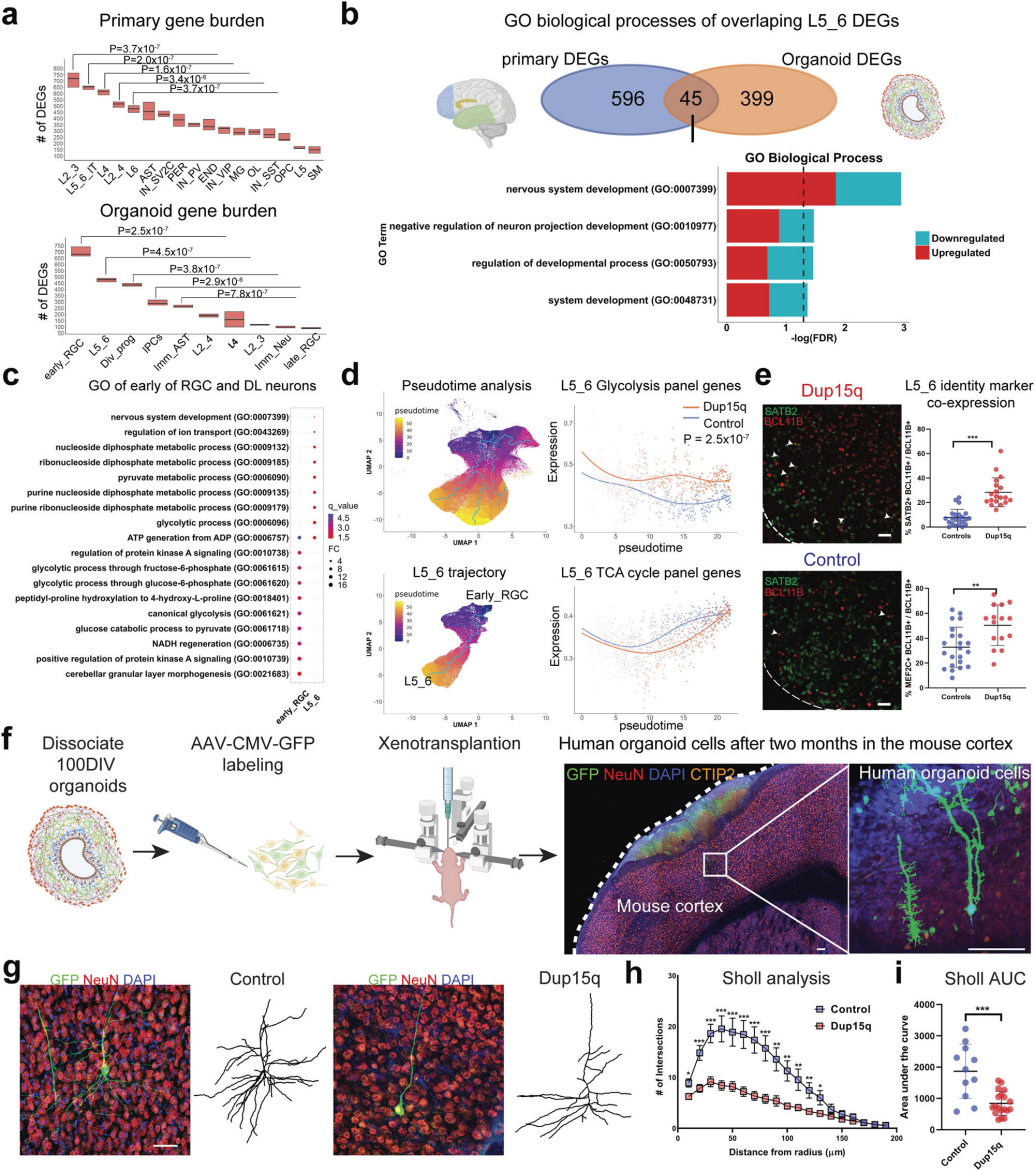
Dup15q deep-layer neurons exhibit increased glycolysis, degraded neuronal-layer identity, and aberrant morphology. **a** Gene burden analysis of primary and cortical organoid cell types. (Differential gene expression was calculated from random 500 nuclei/cells of the control and dup15q groups per cell-type. This analysis was repeated across 10 permutations. Box plots show the median and interquartile range; no whiskers are displayed; *P* values were calculated by comparing numbers of DEGs between cell types using two-sided Mann-Whitney U test). **b** GO analysis of overlapping primary and organoid cortical deep-layer (DL) neurons DEGs, highlighting negative regulation of neuron projection as a common process. Some elements were created in BioRender. Perez, J. (2025) https://BioRender.com/6z5et7u; https://BioRender.com/rpjlezb. **c** GO analysis of organoid early_RGC and DL neurons overexpressed genes showing enrichment of glycolysis associated terms. **d** Pseudotime analysis identifies developmental trajectories and dynamic gene expression changes along deep-layer (DL) neuron differentiation (left). Log-transformed average expression of glycolysis and TCA cycle gene panels are plotted over pseudotime (right). Individual gene-level *P*-values were calculated using tradeSeq (fitGAM and patternTest, two-sided). Following multiple testing correction, a combined meta *P*-value was computed using the logitp method; *P* = 2.47 × 10^-07^). **e** Immunofluorescence (IF) and co-expression analysis of deep-layer (DL) and upper-layer (UL) neuronal markers in organoids, with quantification. (Scale bar = 100 µm. White arrowheads indicate co-expression of BCL11B and SATB2. Plots show the percentage of co-expressing cells among DL neurons. For SATB2, quantification was based on 22 randomly selected images from three control organoid lines and 19 randomly selected images from three Dup15q organoid lines. For MEF2C, quantification was based on 22 randomly selected images from three control organoid lines and 15 images from three Dup15q organoid lines. All organoids were derived from the same differentiation batch. Data are presented as mean values +/- SD; **P = 0.0026; ***P < 0.0001, two-sided unpaired t-test). **f** Illustration of cortical organoid cell dissociation, AAV labeling, and xenotransplantation (Scale bars = 100 µm). Some elements were created in BioRender. Perez, J. (2025) https://BioRender.com/rpjlezb; https://BioRender.com/zf6ehax; https://BioRender.com/h4hyuuj. **g** IF-staining and deep-layer neurite tracing for Sholl analysis (Scale bar = 50 *μ*m). **h** Sholl analysis of deep-layer neurons. (Data are presented as individual points at mean +/-SEM; *P < 0.05, **P < 0.005; ***P < 0.0005, unpaired two-sided t-test, actual *P* values are presented in Data Source; n = 11 from two lines for controls; *n* = 19 from three lines for dup15q). **i** Sholl area under the curve analysis (Data are presented as mean values +/- SD; *p*-value represents unpaired two-sided t-test, *P* = 0.0001). Some figure elements were created with BioRender. Perez, J. (2025).

To test the degree by which dup15q-associated transcriptional changes affect each cell type, we performed gene burden analysis (Methods). In primary tissue, we found that all excitatory neurons were highly affected. Specifically, UL projection neurons (L2_3) had the largest number of DEGs followed by DL intra-telencephalic projecting neurons (L5_6_IT). In organoids, early radial glia had the largest number of DEGs, followed by DL (L5_6) projection neurons (Fig. 2a). This is consistent with previous studies demonstrating a key point of convergence for ASD implicating both DL and UL glutamatergic neurons^2,5,6^. The high gene expression correlation of organoid DL neurons with all primary glutamatergic neurons and the burden discrepancy between organoid cells and primary nuclei might be attributed to developmental changes captured by our experimental paradigm. It is likely that dup15q-associated molecular changes of UL neurons occur later in development and are not yet manifest in the relatively immature UL neurons in our organoids^26^. In contrast, DL neurons arise early during organoid development and are thus relatively more mature and more susceptible to molecular changes at this stage.

To further interrogate how these early changes of organoid neurons culminate in the transcription dysregulation observed in postmortem samples, we compared organoid transcription factor (TF) changes with their potential target genes in postmortem cells. First, we identified TFs from the list of organoid DEGs. We focused on TFs that were dysregulated in either DL or UL neurons. Next, we used hTFtarget^27^ (a database of human transcription factors and their targets) to predict potential gene targets dysregulated in postmortem samples. We found that 41% of DEGs in UL primary neurons and 50% in DL primary neurons are predicted to be targets of TFs dysregulated in organoids. Interestingly, 60-70% of DEGs in primary excitatory neurons were predicted to be targets of organoid early_RGC TFs. (Supplementary Dataset S6, P = Unadjusted Hypergeometric *P*-value). These findings provide evidence for the vulnerability of glutamatergic cortical neurons in dup15q syndrome across developmental stages and link early changes of progenitor and neuronal TFs to gene dysregulation in postmortem samples.

### Increased glycolysis, degraded neuronal layer identity, and aberrant morphology of dup15q DL neurons

Some of the most prominent DEGs shared between organoids and primary DL cortical neurons are known ASD-risk genes such as the BAF (SWI/SNF) chromatin remodeling complex associated transcription factors, *BCL11A* and *BCL11B*, and the netrin receptor, *UNC5A*, required for axon guidance. Gene ontology (GO) analysis of DL neuron DEGs identified “negative regulation of neuron projection development” as a shared associated biological process that is affected in both organoids and primary dup15q DL neurons (Fig. 2b). To explore how early molecular changes in Dup15q might lead to DL neuron projection developmental deficits, we explored the developmental trajectory of DL neurons beginning with early radial glia cells (early_RGC). We found that organoid early_RGCs and DL neurons share many DEGs (*P* = 9.44E-35, Fisher’s exact test). Gene ontology analysis of overexpressed genes identified glycolysis as a common molecular pathway in both cell types, reflecting overexpression of canonical glycolytic enzymes. We validated this finding using immunohistochemistry in organoids at 100DIV (Fig. 2c, Supplementary Fig. S6a). However, we did not observe GO enrichment terms related to these pathways in primary DL neurons, suggesting that overexpression of glycolysis genes may reflect a transient phenotype of early neuronal development. Because metabolic stress has been reported to be a potential feature of organoid culture^26,28^, we also tested glucose intake by measuring media glucose levels in control and dup15q organoids after 8 and 24 h in culture and found increased glucose uptake in dup15q organoids, which was independent of organoid size and not related to the presence of a necrotic core (Supplementary Fig. S6b). Given potential organoid variability, we also performed differential gene expression analysis for early RGCs and DL neurons after shuffling organoid IDs randomly through a set of permutations (Methods). We found that glycolysis was not enriched in 9/10 permutations (Supplementary Dataset S7), confirming that glycolysis was driven mainly by organoid genotype.

Glycolysis and mitochondrial dynamics are tightly regulated and are critical for proper forebrain development. The transition of neural progenitors from hypoxic anaerobic glycolysis to oxidative phosphorylation (OXPHOS) demarcates neuronal differentiation and migration^29^. This transition is largely dependent on the tricarboxylic acid (TCA) cycle and was shown to be critical for the differentiation and maturation of human cultured neurons^30^. Thus, cellular metabolism program shifts may be necessary for cell fate transition and cellular identity^31,32^ during cortical development, and metabolic dysregulation at early stages may contribute to intellectual disability, epilepsy, and neuropsychiatric disorders, including autism, in later life^33–36^. To test whether glycolysis enrichment is unique to dup15q cells transitioning from radial glia to DL neurons, we performed pseudotime analysis in organoids (Methods). We inferred organoid trajectories that faithfully recapitulated in vivo lineage progression (Fig. 2d) and identified differentially expressed genes between control and dup15q organoids in cell-specific lineages along pseudotime (Supplementary Dataset S8). We then plotted the expression of gene sets associated with glycolysis, TCA cycle, and OXPHOS along pseudotime (hallmark and KEGG genes, MSigDB dataset; Methods). We found enrichment in glycolysis concomitant with attenuated TCA cycle expression only within the dup15q DL neuron trajectory (Fig. 2d, Supplementary Fig. S6c). Previous studies have shown that degradation of neuronal identity is associated with increased glycolytic metabolism in organoids^17,26^. Indeed, GO analysis of organoid DL neurons highlights dysregulation of neuron differentiation, regionalization, and pattern specification (Fig. 2b). To test whether glycolysis is associated with cell fate or cell identity anomalies in dup15q DL neurons, we explored the expression of well-referenced DL and UL lineage markers in organoid L5_6 cells. We found that dup15q L5_6 neurons had decreased expression of canonical DL markers (*FEZF2*, *NFIB*, *BCL11B*, and *NEUROD6*) together with increased expression of immature (*DCX* and *PTN*) and UL markers (*SATB2* and *MEF2C*) (Supplementary Fig. S6d). To validate this finding, we performed immunostaining of 100 DIV organoids and found an increased co-expression of DL and UL markers in dup15q L5_6 cells compared to controls (Fig. 2e). In primary DL neurons, we found that many DEGs controlling neuronal projection and identity (Fig. 2b) such as *FOXP2*, *SOX5*, *ETV1*, *BCL11B* and *NRP1* were dysregulated, reflecting a more subtle neuronal identity degradation compared to organoids (mostly relating to DL intratelencephalic vs pyramidal tract identity, Supplementary Dataset S3). Together, these findings suggest that increased glycolysis in dup15q DL neuronal lineage may lead to degraded layer identity.

Given the observed changes in DL marker identity and DEGs associated with neuron projection and morphogenesis, we sought to functionally validate potential DL morphological changes in vitro and after xenotransplantation. We thus cultured organoids up to 100DIV when they are enriched in DL neurons. After dissociation, cells were further matured for two weeks in 2D cultures or labeled and transplanted into the cortex of immunodeficient NSG neonatal mice for two months. We used sparse labeling, confocal imaging, and neurite tracing to assess neuronal morphology by Sholl analysis. We found that dup15q DL neurons had less arborization in both experimental modalities, displaying immature and aberrant morphology (Fig. 2f–i, Supplementary Fig. S7a–c). Morphological differences were more prominent in xenotransplanted neurons compared to neurons in 2D culture. Together, this uncovers a vulnerability of DL projection neurons in dup15q manifested by increased glycolysis during early development, transcription dysregulation of genes associated with neuron projection (shared in organoids and primary tissue), and aberrant morphology.

### Spatially resolved transcriptomic analysis of dup15q tissue sections

**Fig. 3:**
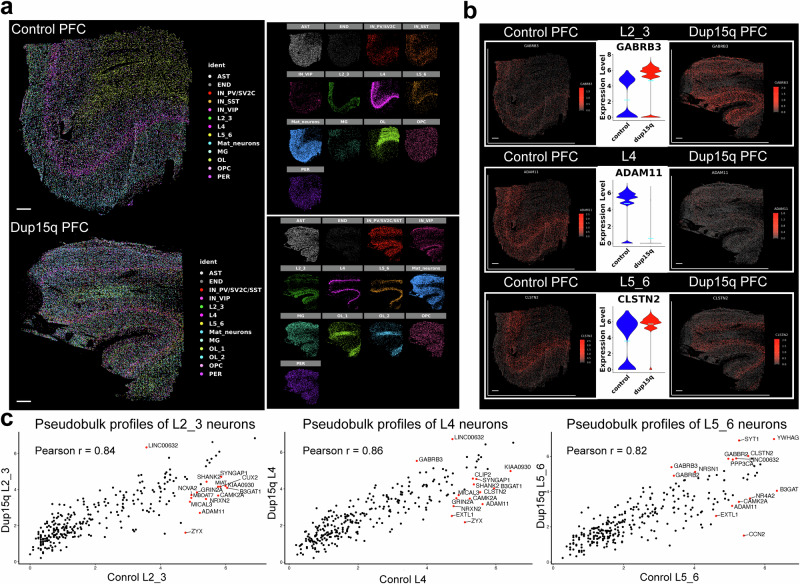
Spatially resolved transcriptomics of the dup15q syndrome prefrontal cortex. **a** Annotated cell clusters overlaid on tissue images using cell coordinates (left; Scale bar = 1 mm). Individual cell type-specific channel images show comparable cell type identification and spatial localization of dup15q patient and control clusters (right). **b** Examples of cell-type-specific differential gene expression validated by spatially resolved transcriptomics (Scale bar = 1 mm). **c** Correlation of spatial transcriptomic gene expression profiles for excitatory neurons. The top differentially expressed genes are in red (*p*-values were calculated using two-sided Wilcoxon Rank Sum and adjusted for multiple comparisons using Benjamini–Hochberg (BH) method. *P*-value < 0.01; Actual *P* values reported in Source Data).

We next explored spatial cell-specific gene expression changes in dup15q brain tissue using a curated list of 285 genes (31 cell identity markers and 254 differentially expressed genes; Supplementary Dataset S9). Using the MERSCOPE platform, we analyzed the spatial expression of targeted genes at sub-cellular resolution in the prefrontal cortex of dup15q and control samples. Pre-processed spatial data was piped into Seurat (v5) for dimensionality reduction, single-cell clustering, and annotation. The analysis identified excitatory neurons from all cortical layers and subtypes of interneurons, glia, and mural cells (Supplementary Fig. S8a, b). Using spatial coordinates, annotated clusters were plotted over the tissue image (Fig. 3a). Despite lower RNA detection rates, we did not observe cellular organization anomalies in the dup15q sample (Fig. 3a, Supplementary Fig. S9a). However, we validated many differentially expressed genes identified initially by snRNA-seq that were highly transcribed across multiple cell types (Supplementary Fig. S9b) and cell-type specific changes at single-cell resolution (Fig. 3b, c). For instance, we observed *GABRB3* as a top overexpressed gene in both UL and DL neurons, *ADAM11* in L4 neurons, and the synaptic regulators *CLSTN2* and *SYNGAP1* in UL neurons (Fig. 3e). Overall, we observed good correspondence of affected cell types between our snRNA data and MERSCOPE transcriptomic datasets in the UMAP embedding space after data integration based on canonical correlation analysis (Supplementary Fig. S9c).

### Convergence of synaptic gene dysregulation between dup15q and idiopathic ASD

Hypothesizing that dup15q gene expression dysregulation coalesces to known ASD-associated genes and pathways, we analyzed the intersection between all primary and organoid DEGs with the list of 1,031 high-risk ASD-associated genes from the Simons Foundation Autism Research Initiative (SFARI) database^37^ (rank 1-3 + syndromic, *N* = 1031; 1/11/22 release). Of 3635 unique primary DEGs, 359 were found in the SFARI gene database (*P* = 1.41E-45, hypergeometric test). Of 1657 unique organoid DEGs, 151 genes intersected with the SFARI dataset (*P* = 1.40E-14, hypergeometric test, Supplementary Fig. S9d). This suggests that dup15q gene dysregulation may involve known ASD genetic pathways. Next, we analyzed this intersection with SFARI genes in specific cell types. In primary dup15q tissue, we found that SFARI genes were enriched in DEGs of excitatory neurons, particularly in UL neurons (L2_3), suggesting that the dup15q ASD pathophysiology is preferentially associated with DEGs in these cell types. In organoids, immature neurons and early radial glia had the highest overrepresentation of SFARI genes (Supplementary Fig. S9e, Supplementary Datasets S3, 4). Organoid DL neurons (L2_3) had the least number of SFARI overlapping genes, again likely reflecting the relatively immature transcriptomic state of organoid UL neurons compared to primary nuclei from patients over 8 years of age^26^.

**Fig. 4:**
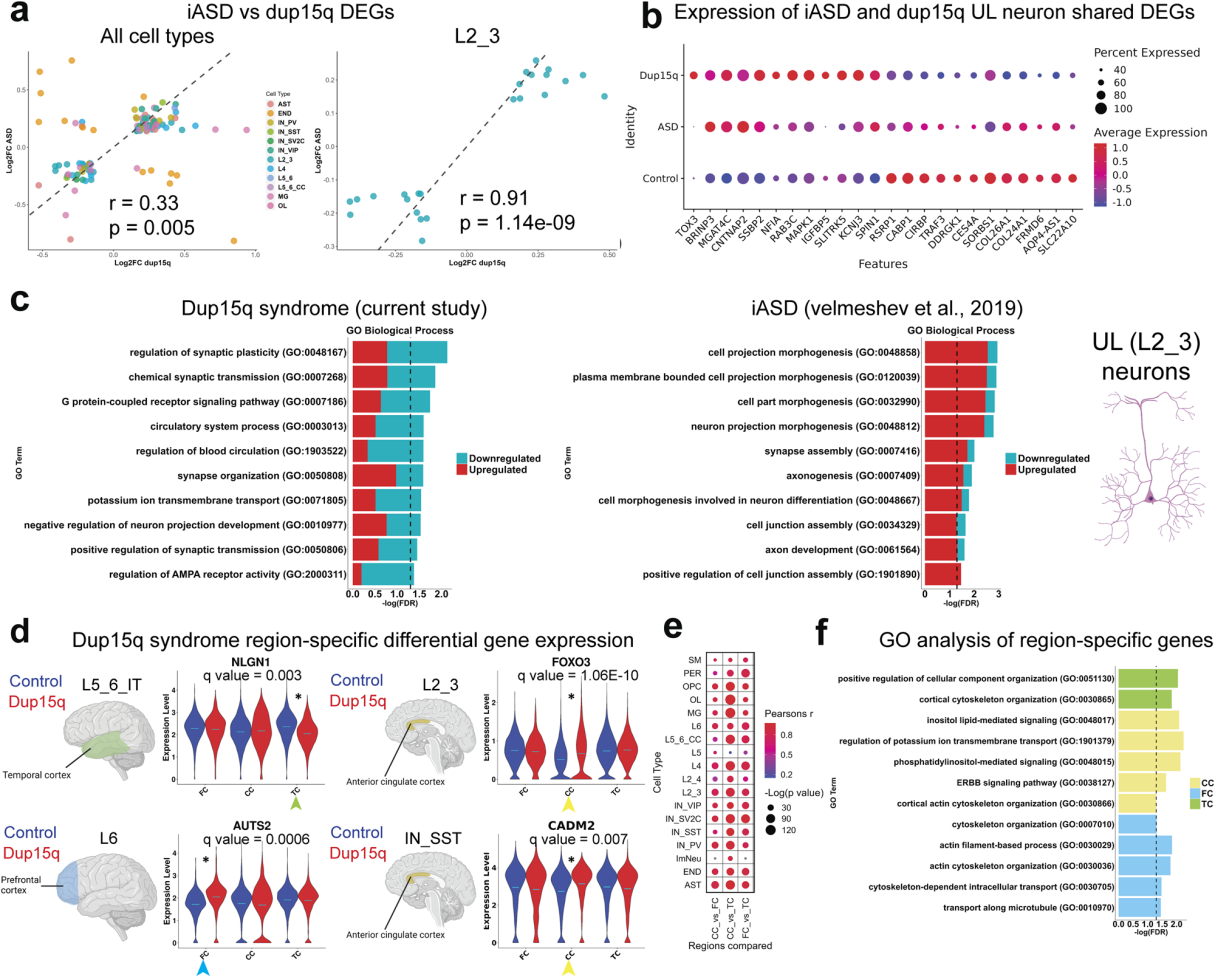
Converged molecular changes of UL neurons between dup15q syndrome and idiopathic ASD. **a** Pearson’s correlation (two-sided) was used to assess the linear relationship between gene expression changes in Dup15q and idiopathic ASD (iASD) across all cell types (left) or between upper layer neurons alone (right). Pearson’s r and *p*-values are shown. Only genes passing FDR < 0.05 were included. Comparisons of DEG fold changes between idiopathic ASD (iASD) and dup15q syndrome across cell types together (left) and for UL neurons separately (right). (r = Pearson coefficient, p = Pearsons *p* value). **b** Differential expression of idiopathic ASD (iASD) and dup15q syndrome shared genes in UL cortical neurons. **c** GO analysis of idiopathic autism (iASD) and dup15q syndrome UL neuron DEGs. Some elements were created in BioRender. Perez, J. (2025) https://BioRender.com/tinyjx3. **d** Violin plots of selected dup15q region-specific gene expression q values represent BH corrected False-discovery rates. Some elements were created in BioRender. Perez, J. (2025) https://BioRender.com/6z5et7u. **e** Pearson’s correlation coefficients were calculated to assess the similarity of differential gene expression (log2 fold changes) between cortical regions for each cell type within the Dup15q vs. control comparison. All correlations were evaluated using a two-sided test, exact *p*-values are reported in Supplementary Dataset S10. **f** GO analysis of dup15q region-specific genes. Some figure elements were created with BioRender. Perez, J. (2025).

Next, we explored the potential convergence of cell-specific molecular changes between dup15q and idiopathic ASD (iASD). Initially, we computed the Pearson’s correlation coefficient for highly expressed genes between corresponding clusters of the dup15q postmortem samples and our previously published iASD snRNAseq datasets^5^. We found a high degree of gene expression correlation between corresponding clusters, showing that annotated cells from each dataset are comparable (Supplementary Fig. S9f). Remarkably when we compared cell-specific fold changes between iASD and dup15q syndrome, we observed significant and cell-type-dependent concordance in gene expression changes between dup15q and idiopathic ASD (Fig. 4a), suggesting overlapping transcriptional programs, particularly within neuronal populations. We found the highest correlation between UL neurons that are the most preferentially affected cell type in both idiopathic iASD and Dup15q syndrome (Fig. 2a)^5^. We found 24 overlapping DEGs (*P* = 3.59E-11, Fisher’s exact test; Supplementary Dataset S3) between iASD and dup15q UL neurons with 23/24 genes showing the same expression trends with a significant degree of gene expression correlation (Pearson’s R coefficient = 0.91, *P* = 1.1E-09; Fig. 4a). Overlapping genes were found to be associated with synaptic function and plasticity (*SLITRK5*, *CABP1*, *MAPK1* and *NFIA*) and with dendritic spine morphology (*CNTNAP2* and *CABP1*) (Fig. 4b). Indeed, GO analysis of iASD and dup15q UL (L2_3) neurons showed enrichment for convergent pathways involving regulation of neuron projection and synaptic-related terms. Many of the observed overlapping genes were upregulated (Fig. 4b, c). These upregulate genes may relate to the finding that neurite complexity appears to be decreased in dup15q (Fig. 2g, h). Of note are upregulated genes involved in the MAPK/ERK signaling pathway (*MAPK1*, *NFIA*, *CNTNAP2*, *IGFBP5*) that are known to be involved in neuronal differentiation and axon growth. Dysregulation of the MAPK/ERK pathway is implicated in autism pathogenesis and causes neurodevelopmental disorders known as “Rasopathies”. It has been shown that an increase in MAPK signaling during corticogenesis can cause aberrant cortical cytoarchitecture and reduced axonal arborization^38,39^. Notably, both neuron projection and synaptic function-related terms have been consistently reported to be affected in postnatal ASD brains, highlighting conserved cell type-specific molecular changes in UL projection neurons between dup15q and other ASD subtypes^2,5,7^.

### Cortical region-specific gene expression changes in dup15q

We next explored cell type-specific dup15q gene expression changes across different brain regions (q value < 0.05; expression fold change ≥ 10%). We found 472 genes across all cell types that were differentially expressed between dup15q and controls in a region-specific manner. The majority of region-specific DEGs were found in PFC (262 genes; 55.5%), followed by TC (111 genes; 23.5%) and ACC (99 genes; 21.0%). Most region-specific DEGs occurred in excitatory neurons (230 genes, 48.7% of all region-specific genes), reflecting spatial heterogeneity of dup15q molecular changes within excitatory subtypes. Only 11 region-specific genes were strong enough to drive the overall cell type-specific DEGs across all regions (6 in neurons and 5 in non-neuronal cells; Supplementary Dataset S10). Some cell-type region-specific genes we found were known ASD-associated genes (Fig. 4d). For example, in L5_6_IT neurons, *NLGN1* was downregulated in the temporal cortex of dup15q but not in the PFC or the ACC. This ASD-associated gene encodes a cell-adhesion protein known for its role in synapse formation and long-term potentiation^40^. In L6 corticofugal projection neurons, *AUTS2* was upregulated in the cingulate cortex (CC). This known ASD risk gene encodes a component of a polycomb group (PcG) multiprotein PRC1-like complex that is important for axon and dendrite elongation through regulation of neuronal gene expression during brain development^41^. *CADM2*, another ASD-associated gene that encodes an adhesion molecule important for synapse organization, was enriched in somatostatin interneurons (IN_SST) in the CC of dup15q. We also found that *FOXO3*, an essential determinant of neuronal morphogenesis and neuronal survival^42,43^, was enriched in dup15q L2_3 projection neurons of the ACC. In addition, we calculated differential gene expression changes for each cortical region separately. We found a good correlation across cell types when comparing cell type-specific DEGs between regions. Grossly, cell-specific DGEs from the CC correlated better with DGEs from the TC (CC_vs_TC, Fig. 4e). Also, we found that immature neurons (Imm_Neu) and subcortical L5 projecting neurons (L5) were the least correlated cell types between regions (Fig. 4e). Gene ontology (GO) analysis showed enrichment for cortical cytoskeletal organization in all cortical regions (Fig. 4f). This might suggest that dup15q region-specific gene expression is implicated in cellular disorganization or cytoarchitectural anomalies in these cortical regions. A previous publication showed neuronal soma volume deficits in the limbic system and other sub-cortical regions of dup15q patients^44^. However, our spatially resolved transcriptomic analysis of dup15q failed to identify such anomalies in the PFC (both qualitatively and by measuring nuclei mean volume as a proxy; Supplementary Fig. S9g).

### Gene co-expression analysis highlights conserved co-expression modules between primary and organoid neurons

**Fig. 5:**
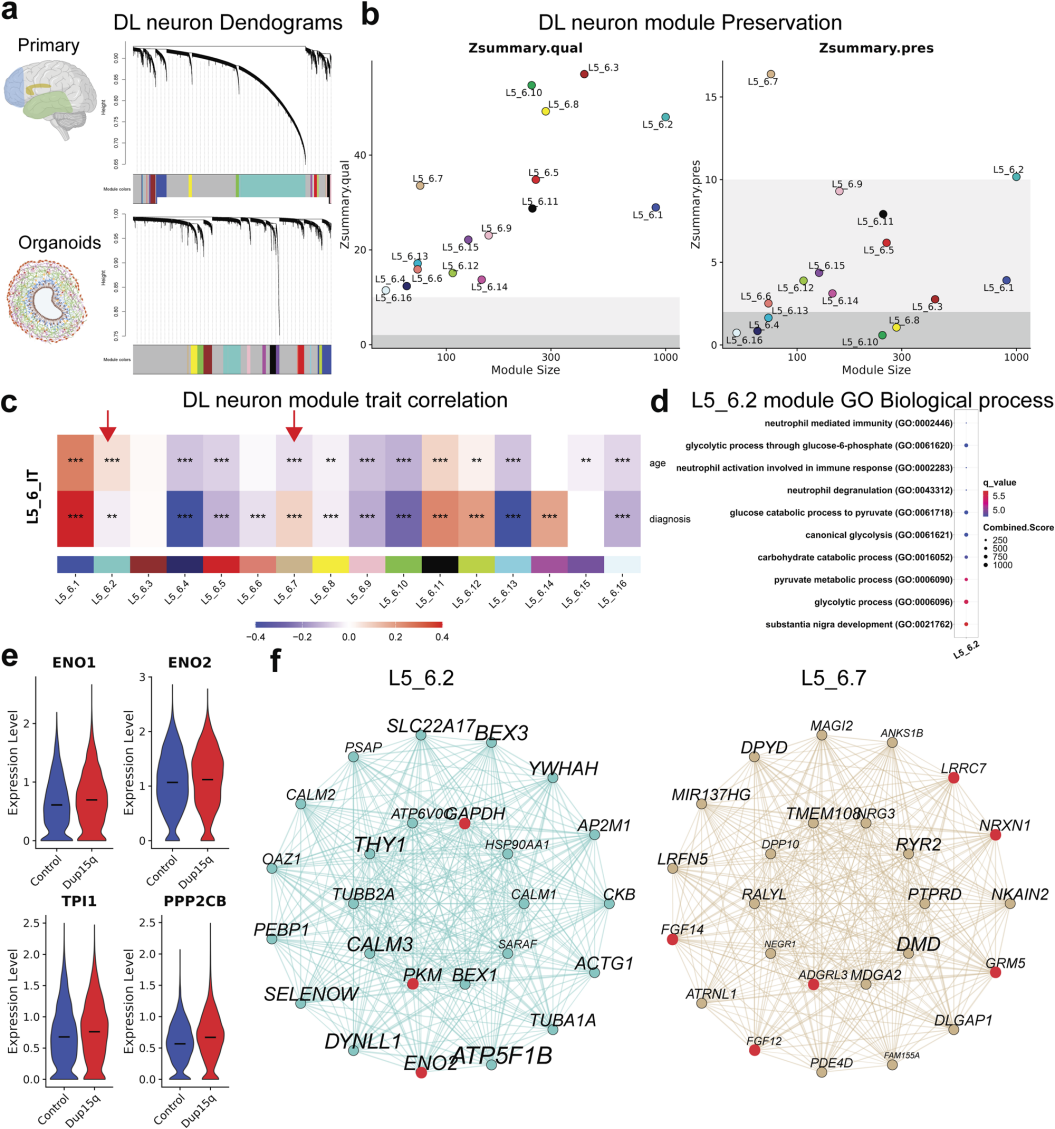
Weighted gene co-expression networks (WGCNA) of organoid and primary dup15q DL neurons. **a** WGCNA dendrograms of primary and organoid deep-layer (DL) neurons. Each leaf represents a single gene, and the colors on the bottom represent the assignment of co-expression modules. Some elements were created in BioRender. Perez, J. (2025) https://BioRender.com/6z5et7u; https://BioRender.com/rpjlezb. **b** Module preservation analysis of primary DL neurons. (Zsummary.qual scores >10 indicate high robustness of module quality; Zsummary.pres >10 indicating strong preservation of the primary modules in the organoid network). **c** Module trait correlation analysis of primary DL neuron modules. The red arrows indicate down or upregulated well-preserved modules (L5_6.2 and L5_6.7) association with the dup15q genotype. Pearson correlation was used to assess the relationship between module eigengenes and Dup15q diagnosis. *p*-values were calculated using a two-sided test. Asterisks denote *p* value significance levels *p < 0.05, **p < 0.01, and ***p < 0.001. **d** GO enrichment analysis of L5_6.2 module, showing enrichment for glycolysis pathways. **e** Violin plots, showing the moderate upregulation of L5_6.2 module glycolysis genes in primary DL neurons. **f** Module network plots of primary DL neurons highlight each network’s top 25 hub genes. Gene font size was scaled to represent each gene’s eigengene-based connectivity (kME). Hub genes associated with glycolysis (L5_6.2) and synaptic functions (L5_6.7) are in red. Some figure elements were created with BioRender. Perez, J. (2025).

To further explore specific pathways dysregulated in dup15q syndrome, we performed high-dimensional weighted gene co-expression network analysis (hdWGCNA) on comparable neuronal types of the postmortem and organoid datasets. Since the datasets shared a high gene expression burden, we focused on excitatory intratelencephalic (IT) neurons and constructed co-expression networks for both DL (L5_6_IT) and UL (L2_3 and L2_4) neurons in the postmortem and organoid samples. We then used the primary neuron co-expression networks as a reference to explore module-level preservation in the organoid query networks. The analysis identified 16 modules in primary DL neurons (Fig. 5a, Supplementary Dataset S11) and 13 in UL neurons (Supplementary Fig. S10a, Supplementary Dataset S12). When compared through module preservation analysis, all organoid DL and UL neuron modules were of high quality in terms of structure and connectivity and were very well-defined in the query network (organoids) compared to the reference (primary tissue) (Zsummary.qual > 10). Most modules of primary DL and UL neurons showed moderate to high preservation scores (Zsummary.pres >5) when compared to organoids (Fig. 5b, Supplementary Fig. S10b), indicating a strong correlation between these modalities at the gene network level. In primary UL neurons, only one module, L2_4.2 was highly preserved (Zsummary.pres > 10, Supplementary Fig. S10a). This module was found to be downregulated in dup15q samples by module trait correlation analysis (Supplementary Fig. S10c). L2_4.2 module was associated with intracellular pH homeostasis, endosomal-lysosomal function, and mitochondrial metabolism (Supplementary Fig. S10d). These biological processes are deeply interconnected with oxidative phosphorylation (OXPHOS), and their downregulation implies energy deficits that may cause synaptic dysfunction. This again, points to potential metabolic reprogramming dysregulation in dup15q syndrome that may lead to neuronal energy deficits. Within DL neurons, two primary modules were highly preserved (Zsummary.pres > 10) in the organoid network (modules L5_6.2 and L5_6.7). Remarkably, module L5_6.2 was found to be associated with glycolysis pathways (Fig. 5d), a hallmark finding potentially influencing organoid DL neuron identity and morphology in organoids. In contrast to our findings in organoid DL neurons, we did not find evidence for the enrichment of glycolysis at the module level in primary neurons (Fig. 5c). Although not statistically significant in our DGE analysis, we did find evidence for a moderate increase of many specific canonical glycolysis genes within the L5_6.2 module (Fig. 5e). This discrepancy may stem from differences in developmental stages, with glycolysis being more active during early brain development and declining as the brain matures. It remains unclear how and to what degree this early metabolic burden culminates in aberrant neuron projection, identity, and morphology in vivo. The other highly conserved DL neuron module we identified, L5_6.7, was associated with ion transport, channel activity, and synaptic organization. This module was enriched in dup15q syndrome (Fig. 5c) and included many synaptic regulators such as *NRXN1*, that regulates neurotransmission and formation of synaptic contacts, *LRRC7*, a regulator of synaptic spine architecture, *ADGRL3*, a member of the latrophilin subfamily of G-protein coupled receptors (GPCR) that plays a critical role in the development of glutamatergic synapses, *GRM5*, a metabotropic glutamate receptor, and *FGF12* and *FGF14*, growth factors known to positively regulate voltage-gated sodium channel activity. Studies of dup15q mouse models and patient stem cell-derived neurons, demonstrate synaptic impairments leading to hyperexcitability^45–47^. Enrichment of the L5_6.7 module in DL neurons of dup15q may point to a specific cluster of genes acting together to contribute to glutamatergic neuron hyperexcitability and seizure phenotypes. Overall, this analysis identified conserved co-expression networks between primary and organoid DL and UL neurons. It highlights a highly preserved glycolysis-associated module, a synaptic-associated module in primary DL neurons, and a mitochondrial metabolism-associated module in UL neurons. These findings support that glycolysis is a robust phenotype consistently preserved across both in vitro and in vivo systems. They also highlight specific gene networks that may contribute to metabolic reprogramming dysregulation in dup15q syndrome, potentially affecting neuronal projection, synaptic function, and the neuronal hyperexcitability observed in dup15q patients (Fig. 5f).

## Discussion

We identify cell-type specific dysregulated gene programs that underlie dup15q pathogenesis and show the vulnerability of excitatory cortical neuron subtypes throughout development. Importantly, we show that dup15q is associated with dysregulation of neuron projection, especially in DL excitatory neurons, which is maintained at postnatal stages. Importantly, we find that a metabolic increase in glycolysis of dup15q progenitors can lead to degraded neuronal identity and aberrant morphology of organoid DL neurons. We show that UL neurons are the most affected cell type postnatally and that changes in organoid neuron TFs could predict the changes of targeted genes in comparable primary neurons, emphasizing that dup15q-associated transcriptional burden is dynamically changing and might correlate with specific cell-type maturation. While we could not find evidence for cellular organization anomalies in the PFC, we were able to validate differentially expressed dup15q syndrome genes at single-cell resolution using spatially resolved transcriptomics. Importantly, we identify cortical region-specific gene changes associated with cytoskeletal organization and highlight brain region-specific changes in dup15q. Furthermore, we find convergent cell-type specific gene expression changes in dup15q and idiopathic ASD, highlighting changes in synaptic regulation of UL neurons that are shared between syndromic and idiopathic ASD. This may suggest shared molecular changes underlying the autism-associated endophenotype observed in dup15q. Weighted gene co-expression analysis revealed temporal shifts in gene expression within dup15q glutamatergic DL and UL neurons and identified conserved gene modules between postmortem brain tissue and organoid models linked to neuronal metabolic reprogramming and increased excitability. These findings highlight dynamic temporal shifts in the cellular and molecular impact of dup15q during neocortical development. Our data, describing transcriptomic changes across cortical cell types in dup15q, as well as our spatial transcriptomic data, can be viewed and interrogated via an interactive web browser: https://dup15q-cortex-organoids.cells.ucsc.edu

## Methods

All postmortem tissue samples obtained were de-identified and collected with appropriate consent for genomic data sharing in accordance with the Autism BrainNet and the NIH NeuroBioBank guidelines. All human pluripotent stem cells (hPSCs) were obtained and processed as approved by the UCSF Human Gamete, Embryo and Stem Cell Research Committee (GESCR, approval 10-05113). All experiments were performed in accordance with protocol guidelines. Animal experiments were performed in accordance with the UCSF – Institutional Animal Care & Use Committee (protocol AN1883074-01D).

### Cortical tissue sample collection and preparation

Dup15q snap-frozen post-mortem tissue and control samples were obtained from the Autism BrainNet bank. Additional control samples were collected from the NIH NeuroBioBank. Cortical samples were sectioned using a cryostat across the entire cortical span (as much as possible) to include grey matter and adjacent white matter. Sections were initially collected (~10 mg) for RNA extraction following an RNA integrity (RIN) assay using 2100 Bioanalyzer and an RNA pico Chip (Agilent). Larger sections (~40 mg) of high quality (RIN > 6.9, unless they were dup15q samples) were collected for nuclei extraction.

### Stem cell characterization maintenance and cortical organoid differentiation

In this work we used the following human induced pluripotent stem cell lines (hiPSC): Dup1-8, Rx68i, SCC115, UCH-CBY4-48B and UCH-PB-48E, obtained and authenticated by the University of Connecticut stem cell core. We also used the 13234 line from the Conklin lab (Gladstone Institute). All stem cell lines used in this study undergone G-banding karyotyping to validate either the dup15q or control genotypes. All lines were karyotyped and analyzed in WiCell (Madison, WI). All control lines showed normal karyotype without any genomic abnormalities or mosaicism. All dup15q lines reports identified the expected abnormality (idic(15q) karyotype for DUP1-8 and Rx68i and interstitial triplication for SCC115). All stem cell lines were validated for pluripotency after receipt. Every 10 passages, stem cells were tested for karyotypic abnormalities and validated for pluripotency markers Sox2, Nanog, and Oct4. All cell lines tested negative for mycoplasma. All hiPSC lines were expanded on growth factor-reduced Matrigel (BD)-coated plates. hiPSCs were thawed in StemFlex Pro Media (Gibco) supplemented with 10 µM Rock inhibitor Y-27632 which was removed the following day. Medium was changed every day and lines passaged when colonies reached 80% confluency. Stem cells were passaged using ReLeSR (Stemcell technologies) and manually lifted with cell lifters (Fisher). All lines used for this study were bellow passage 30. Cortical organoids were cultured using a dorsal forebrain differentiation protocol^16^. Briefly, hiPSC lines were expanded and dissociated to single cells using accutase (Gibco). Cells were then aggregated in neural induction medium at a density of 10,000 cells per well in 96 well v-bottom low adhesion plates. GMEM-based neural induction medium included 20% Knockout Serum Replacer (KSR), 1X non-essential amino acids, 0.11 mg/mL Sodium Pyruvate, 1X Penicillin-Streptomycin, 1X Primocin, 0.1 mM Beta Mercaptoethanol, 5uM SB431542 and 3 µM IWR1-endo. Medium was supplemented with 20 µM Rock inhibitor Y-27632 for the first week. After 18 days organoids were transferred to six well ultra-low adhesion plates and moved to an orbital shaker rotating at 90 rpm. Media was changed to DMEM/F12-based medium containing 1X Glutamax, 1X N2, 1X CD Lipid Concentrate and 1X Penicillin-Streptomycin. At 35 days, organoids were moved into DMEM/F12-based medium containing 10% FBS, 5 µg/mL Heparin, 1X N2, 1X CD Lipid Concentrate and 0.5% Matrigel. At 70 days medium was additionally supplemented with 1X B27 and Matrigel concentration increased to 1%. Organoids were collected for single cell capture and immunohistochemistry at relevant timepoints.

### Nuclei isolation and snRNA-seq

Dup15q and matched control samples were processed as previously described in ref. ^48^. Briefly, sectioned brain tissue was homogenized in 5 mL of RNAase-free lysis buffer (0.32 M sucrose, 5 mM CaCl_2_, 3 mM MgAc_2_, 0.1 mM EDTA, 10 mM Tris-HCl, 1 mM DTT, 0.1% Triton X-100 in DEPC-treated water) using a glass dounce tissue homogenizer (Thomas Scientific, Cat # 3431D76) on ice. The homogenate was loaded into a 30 mL thick polycarbonate ultracentrifuge tube (Beckman Coulter, Cat # 355631). 9 mL of sucrose solution (REF) (1.8 M sucrose, 3 mM MgAc_2_, 1 mM DTT, 10 mM Tris-HCl in DEPC-treated water) was added to the bottom of the tube with the homogenate and centrifuged at 107,000 g for 2.5 h at 4 °C. Supernatant was aspirated, and nuclei pellet was incubated in 250 uL of DEPC-treated water-based PBS for 20 min on ice before resuspending the pellet. Nuclei were filtered 3 times to remove debris using a 30 *μ*m pre-separation filters (Miltenyibiotec). Nuclei were counted using a hemocytometer and diluted to 1000 nuclei/uL before performing single-nuclei capture on the 10X Genomics Single-Cell 3’ system. Target capture of 10,000 nuclei per sample was used. Nuclei capture and library preparation was done according to the Chromium Single Cell 3’ Reagent Kits User Guide V3 (10x genomics). Single-nuclei libraries were pulled and sequenced as per manufacturer recommendation on a NovaSeq S2 flow cell.

### Cortical organoid single cell capture and scRNA-seq

Organoids were collected for single cell capture and immunohistochemistry (N = 3 organoids per line per timepoint) at 50, 100 and 150 days of in-vitro development (DIV). Organoids were dissociated using papain (Worthington) solution containing DNase. Samples washes 3 times in 1XPBS, placed in 1.5 ml papain and incubated at 37 °C. tubes were inverted every 15 min for up to a total of 45 min. Next, samples were triturated by manually pipetting with a glass pasteur pipette approximately until homogenized. Dissociated cells were spun down at 300 g for 5 min and papain removed. Similar to nuclei capture, 10,000 cells per sample was used and library preparation was done according to the Chromium Single Cell 3’ Reagent Kits User Guide V3 (10x genomics). Single-cell libraries were pulled and sequenced on a NovaSeq S2 flow cell.

### 10X Genomics CellRanger software and data filtering

CellRanger software v 6.1.1 was used for library demultiplexing, fastq file generation, read alignment and UMI quantification. CellRanger was used with default parameters using the GRCh38-2020-A transcriptome reference, while using the “include-introns” option as pre-mRNA reference file for nuclei data. These were piped into Seurat v.4. For nuclei analysis^45,46^, expression matrices containing numbers of Unique molecular identifiers (UMIs) per nucleus per gene were filtered to retain nuclei with at least 500 genes expressed and less than 5% of total UMIs originating from mitochondrial and ribosomal RNAs (nCount_RNA > 500, percent.mt <5% and percent.ribo <5%). Individual matrices were combined, UMIs were normalized to the total UMIs per nucleus and log transformed.

For organoid cell analysis expression matrices containing numbers of UMIs per cell per gene were filtered to retain cells with at least 1000 genes expressed and less than 10% of total UMIs originating from mitochondrial and 25% ribosomal RNAs (nCount_RNA > 1000, percent.mt <10% and percent.ribo <25%).

### Dimensionality reduction, clustering, cell type annotation and UMAP visualization

Data preprocessing, normalization, variable feature selection and PCA was performed using the standard Seurat (v.4.) pipeline^45,46^. Briefly, filtered log-transformed UMI matrix was used to perform principal component analysis. Scree plot was generated to select the number of significant principal components (PCs) by localizing the last PC before the explained variance reaches plateau. Using this approach, k = 10 nearest neighbours were used for both postmortem nuclei and organoid cells to calculate Jaccard distance-weighted nearest neighbor distances which was used to perform Louvain clustering. To visualize transcriptomic profiles in two-dimensional space, we used Uniform Manifold Approximation and Projection for Dimension Reduction (UMAP) technique. The identity of specific cell types was determined based on expression of known marker genes, as shown in Fig. 1 and figure S2c, d. One primary nuclei cluster that was previously identified as neurogranin (NRGN) expressing neurons^48^ was annotated as neuronal debris due to nuclear-retained transcript depletion and high content of mitochondrial gene expression^47^ (Figure S2e, f).

### Differential gene expression analysis

We downsampled the data so that the control and dup15q groups across all cell types had the same number of nuclei/cells. To identify genes differentially expressed in dup15q samples for both primary nuclei and organoid cell-types we used MAST to perform zero-inflated regression analysis by fitting a linear mixed model (LMM)^24^. To exclude gene expression changes stemming from confounders, such as age, sex, RIN, cortical region, 10X capture batch and sequencing batch, the following model was fit with MAST for primary nuclei:

zlm(~diagnosis + (1|ind) + cngeneson + age + sex + region + RIN + PMI + Capbatch + Seqbatch + ribo_perc, method = “glmer”, ebayes = F, silent=T, parallel = T)

and for organoid cells:

zlm(~diagnosis + (1|ind) + cngeneson + DIV + line + sex + source + reprograming + karyotype + Capbatch + Seqbatch + ribo_perc, method = “glmer”, ebayes = F, silent=T, parallel = T).

Where cngeneson is gene detection rate (factor recommended in MAST tutorial), Capbatch is 10X capture batch, Seqbatch is sequencing batch, ind is individual label, PMI is postmortem interval, DIV is days of in-vitro differentiation, source is the source tissue cells were reprogrammed from, reprograming is the method used for iPSC generation, ribo_perc is ribosomal RNA fraction, mito_perc is mitochondrial RNA fraction and mito_nucl_perc is the fraction of nuclear-encoded mitochondrial RNA. When testing for differential expression in each cluster, the following strategy was used to prevent individual bias from affecting differential gene expression analysis. First, average number of cells contributed by each individual sample to a given cluster was calculated. Then, individuals contributing less than 25% of this average were removed from the analysis. Equal number of cells was selected from each sample, and differential expression was performed. This routine was repeated 10 times to sample most cells in each cluster. On average across clusters, this strategy allowed including 85% of all individuals when performing the analysis while preventing nuclei profiles from few individuals from dominating differential gene expression estimates. Expression fold change cut off was set at 14% to enable comparisons with idiopathic ASD dataset^5^. In addition, we calculated raw fold change of gene expression by repeating MAST analysis with only diagnosis factor in the model and filtered out genes with raw fold change of expression less than 0.1. The latter filtering step allowed removing genes whose fold change of expression was heavily dependent on the confounding factors, rather than clinical diagnosis.

For correlation analysis of the union of genes differentially expressed in either primary or organoid cells (Supplementary Fig. S4d) we used the AverageExpression function in Seurat to calculate fold changes.

### Permutation analysis

To verify the ability of our model to capture true gene expression changes, we preformed random permutation of individual labels in each cluster between control and dup15q groups before performing differential gene expression analysis. This analysis was repeated over 10 permutations. Empirical *p*-values and FDR were derived from the null distribution generated across permutations.

### Burden analysis

To calculate number of dup15q-associated DEGs across cell types and normalize by number of cells in each cluster, we randomly drew 500 nuclei/cells from the control and dup15q groups and each cell type before performing differential expression analysis. This analysis was repeated across 10 permutations, and average number of DEGs for each cell type was estimated.

### Trajectory reconstruction and identification of lineage-specific dynamically expressed genes in the organoid dataset

Seurat UMAP coordinates were imported into monocle3^49^ for trajectory reconstruction. learn_graph function with custom graph_control options were used to construct the trajectory graph. To focus on specific lineages, we isolated the shortest path between the node in the neural progenitor/radial glia cluster and the node in the more mature cell type clusters. Then, we selected cells along the trajectory branches corresponding to specific lineages. After that, monocle3’s Moran’s test (graph_test function) was used to identify genes that are dynamically expressed in each lineage in control and dup15q organoids separately. We modified graph_test function to utilize Moran’s test with covariates to ensure that our results are not affected by uneven contribution of cells from controls and Dup15q subjects, as well as cells from different iPS cell lines. We selected genes with adjusted *p*-value < 0.05 and Moran’s I > = 10 as statistically significant dynamically expressed genes. To identify lineage-specific genes, we first compressed the single-cell expression data along each lineage by using a sliding window along pseudotime and averaging expression of neighboring cells for each gene. We generated 500 meta-cells in each lineage using this approach.

### Identification of dynamically expressed genes dysregulated in dup15q organoids in specific lineages

To identify dynamically expressed genes dysregulated in dup15q organoid lineages compared to controls, we selected all genes dynamically expressed in control and dup15q organoids in each lineage. Then, for each lineage and gene, we calculated the difference of the areas under the curve for the smoothed expression/pseudotime plot for each gene in each lineage between control and dup15q and termed it differential expression score. All genes with the differential expression score of at least 50 were considered differentially expressed between control and dup15q. For plotting combined gene expression for all genes in specific pathways (glycolysis, oxidative phosphorylation, citric acid cycle), we used curated gene sets from MSigDB^50^ (HALLMARK_GLYCOLYSIS, HALLMARK_OXIDATIVE_PHOSPHORYLATION and KEGG_CITRATE_CYCLE_TCA_CYCLE). We then calculated averaged gene expression of all genes in a pathway in each meta-cell and each lineage separately for control and dup15q organoids. Log-transformed values of averaged expression were plotted over pseudotime. We performed the comparison of the control and dup15q L5_6 lineages, individual *p* values for each gene were calculated using tradeSeq fitGAM and patternTest. After multiple comparison adjustments, meta *p* value was calculated using metap package’s allmetap function and the logitp method.

### **Organoid size and glucose intake assay**

Organoids were cultured up to 50DIV, a stage in which organoids are enriched with early_RG and DL neurons. For size measurements, we took images with an inverted phase contrast microscope (Olympus CKX53) and analyzed organoid surface area via imageJ. For glucose assay, individual organoids were placed a single well of a 48-well plate with 100 ul of media (just enough to cover the organoid) and were left inside the incubator (on shaker). Media was collected after 8 and 24 h for glucose measurements. Media was diluted 1:100 in PBSx1 and glucose levels were quantified using the Glucose-Glo™ Assay (Promega) according to manufacturer’s instructions.

### Region-specific differential gene expression analysis

To test for age and diagnosis interaction, the following LMM model was fitted:

zlm(~diagnosis:age + diagnosis + sequencer + (1|ind) + cngeneson + age + sex + RIN + region + Capbatch + Seqbatch + ribo_perc + mito_perc + mito_nucl_perc, sca, method = “glmer”, ebayes = F, silent=T) And LRT test was performed after removing diagnosis:age interaction term from the model. Genes with FDR < 0.05 were considered as significantly affected by interaction between age and dup15q diagnosis. This analysis was performed for all clusters. For region-specific analysis, nuclei from either the PFC, ACC or TC were selected, and the same LMM model was utilized as when testing for differential gene expression between Dup15q and Control, except for removing the region factor.

### Spatially resolved transcriptomics

Sample preparation was performed according to manufacturer’s instructions (MERSCOPE Fresh and Fixed Frozen Tissue Sample Preparation User Guide, Doc. number 91600002). Briefly, fresh snap frozen tissues from the prefrontal cortex of dup15q syndrome patient and control, having a high RNA integrity number (RIN > 8) were sectioned (10um thick) using a cryostat and mounted on MERSCOPE functional slides. Sections were then fixed and stored at 70% ethanol for up to two weeks. Sections went through autofluorescence quenching under UV light for 3 hours using the MERSCOPE Photobleacher instrument. A pre-designed panel mix (285 genes) focused on highly differentially expressed genes based on the single-nuclei analysis were used for probe hybridization. Hybridizations were performed at 37 °C for up to 48 h in a humid environment. Post prob hybridization, sections were fixed using formamide and embedded in gel. After gel embedding, tissue samples were cleared using a clearing mix solution supplemented with proteinase K for 24–48 h at 37 °C until no visible tissue was evident in the gel. After clearing was completed, sections were stained for DAPI and PolyT and fixed with formamide prior to imaging. The MERSOPE imaging process was done according to the MERSCOPE Instrument Site Preparation Guide, Doc. Number 91500001. Briefly, an imaging kit was thawed at 37 °C for 45 min, activated and loaded into the instrument. MERSCOPE flow chamber was then assembled with the stained tissue section, fluidics were primed, flow chamber filled with liquid and a low-resolution image was taken. Based on DAPI staining, an ROI was chosen for the full imaging experiment. After imaging was complete, data was processed using MERSCOPE proprietary software, while using DAPI and polyT staining for cell segmentation and RNA puncta cellular assignment. Further analysis, visualization, and integration of spatial data was done using Seurat v5 (Source: vignettes/spatial_vignette_2.Rmd).

### High-dimensional weighted gene co-expression network (hdWGCNA)

To construct hdWGCNA network from single nuclei/cell RNA-seq data, we used the hdWGCNA R package^51^. Initially, we subset the Seurat object to include DL or UL excitatory neurons from nuclei and all excitatory cells from organoids. We then set up a Seurat object for WGCNA by selecting genes that are expressed in at least 5% of cells in each dataset. We continued by constructing metacells using k-Nearest Neighbors (KNN = 25) to yield a metacell gene expression matrix. Following normalization and scaling of the data, we tested different softpowers to select for thresholding when performing WGCNA. When constructing co-expression network, we used soft_power=10 for primary neurons and soft_power=8 for organoids. We then computed all module eigengenes (MEs) in the full single-nuclie/cell datasets, harmonized module eigengenes and computed eigengene-based connectivity (kME) for each gene. We also performed module trait correlation to generate a heatmap of disease associated modules and used enrichR^52^ to perform module specific gene ontology enrichment. Finally, we used the ModuleNetworkPlot function to visualize the network underlying the top 25 hub genes for each disease-associated module. Module preservation was performed with the primary neuron co-expression modules as reference using the ModulePreservation function for 100 permutations.

### Dup15q and idiopathic ASD clusters gene expression correlation

To account for library size and number of cells per cluster, normalized average counts of each cluster from each dataset was obtained using Seurat’s AverageExpression function. For comparison, top 20 markers from each cell-type were used. Counts were converted to counts per million (CPM) and log transformed. Correlation analysis and plotting was performed using the corrplot R package.

### Statistical overrepresentation test for Gene Ontology (GO) terms

PANTHER^53^ was used to perform statistical overrepresentation test for DEGs from each cluster. All genes expressed in a given cluster were used as the background and GO Biological Processes ontology was used. Processes with FDR < 0.05 were considered and sorted by FDR.

### Hypergeometric testing

To estimate the significance of the overlap of two gene lists, we performed hypergeometric testing with all detected genes in primary samples as the background list. To estimate overlap of ASD genetic risk factors with DEGs in each cell type, list of DEGs in a given cell type was used as the sample and list of all genes expressed in the cell type as the population list. These lists were overlapped with all genes in the SFARI Gene Module database, genes with ranks 1 to 3 or genes labeled as “syndromic”. Hypergeometric *p*-values were FDR-corrected using Benjamini and Hochberg procedure.

### Histology and immunohistochemistry

Human brain tissue blocks were snap-frozen and stored at −80 °C. 16µm-cryosections were collected on superfrost slides (VWR) using a CM3050S cryostat (Leica) and fixed in 4% PFA at room temperature (RT). Organoids were captured at 50, 100 and 150 DIV, washed with 0.1 M PBS and fixed in 4% PFA at room temperature (RT) for 1 hour. Organoids were then washed with 0.1 M PBS and moved to a solution of 30% sucrose at 4 °C overnight. Organoids were snap-frozen and stored at −80 °C in a cryomold filled with a 1:1 30% sucrose to OCT ratio. For both organoid and brain tissue immunohistochemistry, 16 µm sections were initially treated with heated (90 °C) citrate-based antigen unmasking solution (Vector laboratories) and blocked in 0.1 M PBS/0.1% Triton X-100/ 10% goat/horse/donkey serum for 30 min at RT. Primary antibody incubations were carried out overnight at 4 °C. After washing with 0.1 M PBS, cryosections were incubated with secondary antibodies diluted in 0.1 M PSB/ 0.1% Triton X-100 for 2 hours at RT. For immunofluorescence, Alexa fluochrome-tagged secondary IgG antibodies (1:500, Invitrogen) were used for primary antibody detection. Slides with fluorescent antibodies were mounted with DAPI Fluoromount-G (SouthernBiotech) and imaged on a Leica TCS SP5 confocal microscope. For intermixed neuron identity analysis, images were analyzed using Imaris x64 9.7.1. SATB2 (depicted in green) and BCL11B (depicted in red) were counted automatically. Overlapping was detected using the Imaris filter “shortest distance to” (lower threshold 0, upper threshold 1.5). Numbers shown are SATB2 and BCL11B double positive cells normalized by CTIP2 positive cells. For PGK1 expression, positive cells were quantified using the imaris spots detection function (minimum diameter threshold 7 and minimum quality filter threshold 11). The plot represents the percentage of PGK1 positive cells relative to DAPI.

### Single-molecule in situ RNA hybridization

Tissue blocks were sectioned using a Cryostat (Leica) at 10 μm onto glass cover slides and stored at −80 °C. For RNA in situ hybridization, the RNAscope Multiplex Fluorescent Reagent Kit v2 was purchased from ACD Advanced Cell Diagnostics and RNAscope probes against UBE3A (Hs-UBE3A; #570691), PLXNA4 (Hs-PLXNA4-C2; #460041-C2) and GABRG3 (Hs-GABRG3-C3; #525691-C3) were purchased from Advanced Cell Diagnostics (ACD biosciences). As fluorophores, Opal 520 (1:500), Opal 570 (1:750) and Opal 690 (1:750) (Akoya Biosciences) were used. For sample preparation, slides were fixed in prechilled 4% PFA for 1 hr at 4 °C and rinsed twice with PBS. Sections were dehydrated in a sequential ethanol gradient of 50%, 70%, 100%, 100% EtOH for 5 min. at RT each. Sections were air dried for 5 mins and a hydrophobic barrier was drawn around each section and dried for 5 min at RT. 5 drops of RNAscope hydrogen peroxide were added to each section and incubated for 10 min at RT. Slides were washed twice with DI water. 5 drops of protease IV were added to each section and incubated for 30 mins at RT. Slides were washed twice with 1X PBS. RNAscope probes were hybridized for 2 h at 40 °C and all subsequent steps were conducted according to the manufacturer’s recommendation (RNAscope Multiplex Fluorescent Reagent Kit v2 User Manual, #323100-USM/Rev Date: 02272019).

### RNAscope Imaging, quantifications, and statistics

Initially, grey and white matter were distinguished using nuclei morphology and immunohistochemical staining for *NEUN* and *MBP*. Images for quantifications of RNA in situ hybridization were focused on regions of grey matter. Images were acquired using a confocal microscope (Leica TCS SP5 X, 63X objective). The investigator was blinded to sample labels before acquiring and processing the images, and all image processing steps were performed with the same parameters for all sections and individuals. Seven fields of view were taken for each sample. All fluorescent pictures are z-stacked. RNA counts were detected and quantified by Imaris software while using the same detection parameters for both control and dup15q images. RNA counts were then normalized to number of nuclei detected by DAPI for each image. For statistical analysis, an unpaired two-tailed student’s t-test was performed using the Graphpad prism 8.0 software.

### **2D culture Sholl analysis**

Organoids were dissociated at 100 DIV using the Papain Dissociation System (Worthington Biochemical) and seeded at a concentration of 200,000 cells per cm2 on laminin and PDL coated coverslips (Neuvitro Corporation). Cells were cultured in maturation medium^54^ for two weeks prior to cell collection (BrainPhys medium supplemented with 1× B27, 1× N2, 20 ng ml−1 BDNF, 20 ng ml−1 GDNF, 1 mM dibutyryl-cAMP, 200 nM ascorbic acid, 1 μg ml−1 laminin and antibiotics). Half of the medium was changed with fresh medium every 3 days. Four days before collection, cells were transfected with 1ug of EGFP-C1 expression vector per well using Lipofectamine 2000 (Invitrogen). Cells were fixed with 4% formaldehyde and 4% sucrose in PBS for 15 min, washed with PBSx1 three times, then permeabilized and blocked with PBS-based blocking buffer containing 10% donkey serum, 0.2% gelatin and 0.1% Triton X-100 at room temperature for 1 h. Samples were then incubated with primary antibody at 4 °C overnight. The next day, samples were washed in PBS three times and incubated with secondary antibody in the blocking buffer at room temperature for 1 h. Samples were then washed in PBS twice, counterstained with DAPI and washed in PBS once more. z-stack images were acquired with a ZEISS LSM 900 Confocal Microscope x20 objective. Neurite detection and Sholl analysis was carried out for BCL11B positive cells using the SNT Sholl analysis plugin in Fiji (imageJ). Radius distance for measurements was set for every 10*μ*m from the soma. Statistical analysis was carried out using GraphPad Prism 8.

### Organoid cell xenotransplant Sholl analysis

Initially, organoids were cultured for 100 days in vitro. At this stage organoids are enriched for deep layer projection neurons. Organoids from two control and three Dup15q lines were dissociated using the Worthington papain dissociation system, washed in PBSx1 and incubated with an adeno-associated virus expressing GFP in a concentration of 1 × 10^4^ GC/cell for 8 hours (AAV1-CMV-GFP; Charles River). Cells were then washed three times with PBSx1 and counted using a hemocytometer. Cells were then immediately immediate transplantation into P4 postnatal NSG mice (NOD.Cg-Prkdc^scid^ Il2rg^tm1Wjl^/SzJ mice, 005557, The Jackson Laboratory) as previously described^26,55^. Newborn mice used for cellular injections (P4, including males and females) were anesthetized by hypothermia on ice for 2–3 min. The pups were then taped onto a head mold and placed onto a stereotactic injector. Cells were stereotaxically injected at 1 × 10^5^ human organoid cells per 100 nl. Injections were carried out in each cortical hemisphere via transcranial injection. After 2 minutes, the pipette was retracted and the pups were returned to their mother. Recovery occured within 1–2 min of returning the mice to their cage. Mice were allowed to develop for 2 months before being euthanized. A total of 9 mice were used (5 males, 4 females). After two months, animals were euthanized using Avertin, perfused with 4% PFA before brains were extracted. Brains were then further incubated in 4%PFA, following incubation in 4% sucrose, and OCT embedding. Tissue blocks were sectioned at 100 *μ*m thickness for IHC (as described above), imaging, and Sholl analysis. z-stack images were acquired with a ZEISS LSM 900 Confocal Microscope x20 objective with a 1 µm intervals. Neurite detection and Sholl analysis was carried out for BCL11B positive cells using the SNT Sholl analysis plugin in Fiji (imageJ). Radius distance for measurements was set for every 10 *μ*m from the soma. Statistical analysis was carried out using GraphPad Prism 8. Researchers were blind to sample IDs during neurite tracing.

### Antibodies

**Table:** 

Antigen	Host species	Manufacturer	Identifier	Dilution
MBP	goat	Santa cruz bio	SC-13914	1:500
SOX2	Mouse	Santa cruz bio	sc-365823	1:500
EOMES	Sheep	R&D systems	AF6166	1:500
BCL11B	Rat	Abcam	ab18465	1:500
HOPX	Rabbit	Proteintech	11419-1-AP	1:500
SATB2	mouse	Abcam	ab51502	1:500
NeuN	Guinea pig	Millipore	ABN90	1:500
AQP4	Rabbit	Proteintech	16473-1-AP	1:500
PGK1	Rabbit	ThermoFisher	PA5-13863	1:100
MEF2C	Rabbit	Cell Signaling	D80C1	1:500

Some figure elements were created in BioRender. Perez, J. (2025).

### Reporting summary

Further information on research design is available in the Nature Portfolio Reporting Summary linked to this article.

## Supplementary information

Supplementary Information

Description of Additional Supplementary Files

Supplementary Dataset S1

Supplementary Dataset S2

Supplementary Dataset S3

Supplementary Dataset S4

Supplementary Dataset S5

Supplementary Dataset S6

Supplementary Dataset S7

Supplementary Dataset S8

Supplementary Dataset S9

Supplementary Dataset S10

Supplementary Dataset S11

Supplementary Dataset S12

Reporting Summary

Transparent Peer Review file

## Source data

Source Data

## Data Availability

The raw data generated in this study can be accessed at the NCBI Sequence Read Archive (SRA), accession number PRJNA1017130. Analyzed data (cell-count matrix and metadata) can be accessed through the UCSC Cell Browser, collection dup15q-cortex-organoids (https://dup15q-cortex-organoids.cells.ucsc.edu). All other data associated with this study are present in supplementary materials and tables. Additional data associated with this paper is provided in Source Data. Source data are provided with this paper.

## References

[1] Sandin, S. et al. The Heritability of Autism Spectrum Disorder. *JAMA* **318**, 1182–1184 (2017). 28973605 10.1001/jama.2017.12141 PMC5818813

[2] Parikshak, N. N. et al. XIntegrative functional genomic analyses implicate specific molecular pathways and circuits in autism. *Cell* **155**, 1008 (2013). 24267887 10.1016/j.cell.2013.10.031 PMC3934107

[3] Suzuki, K. et al. Microglial Activation in Young Adults With Autism Spectrum Disorder. *JAMA Psychiatry* **70**, 49–58 (2013). 23404112 10.1001/jamapsychiatry.2013.272

[4] Parikshak, N. N. et al. Genome-wide changes in lncRNA, splicing, and regional gene expression patterns in autism. *Nature* **540**, 423–427 (2016). 27919067 10.1038/nature20612 PMC7102905

[5] Velmeshev, D. et al. Single-cell genomics identifies cell type–specific molecular changes in autism. *Sci. (80-).* **364**, 685–689 (2019). 10.1126/science.aav8130 PMC7678724 31097668

[6] Willsey, A. J. et al. XCoexpression networks implicate human midfetal deep cortical projection neurons in the pathogenesis of autism. *Cell* **155**, 997 (2013). 24267886 10.1016/j.cell.2013.10.020 PMC3995413

[7] Voineagu, I. et al. Transcriptomic analysis of autistic brain reveals convergent molecular pathology. *Nature* **474**, 380–386 (2011). 21614001 10.1038/nature10110 PMC3607626

[8] Gandal, M. J. et al. Broad transcriptomic dysregulation occurs across the cerebral cortex in ASD. *Nature* **611**, 532–539 (2022). 36323788 10.1038/s41586-022-05377-7 PMC9668748

[9] Li, C. et al. Single-cell brain organoid screening identifies developmental defects in autism. 1–26 (2022). 10.1038/s41586-023-06473-y PMC10499611 37704762

[10] Quadrato, G. et al. *Autism genes converge on asynchronous development of shared neuron classes*. vol. 602 (Springer US, 2022). 10.1038/s41586-021-04358-6 PMC8852827 35110736

[11] Mariani, J. et al. Glutamate Neuron Differentiation in Autism FOXG1-Dependent Dysregulation of GABA / Glutamate. 375–390 10.1016/j.cell.2015.06.034 (2015). 10.1016/j.cell.2015.06.034 PMC4519016 26186191

[12] Courchesne, E. et al. Neuron Number and Size in Prefrontal Cortex of Children With Autism. **306**, 2001–2010 (2011). 10.1001/jama.2011.1638 22068992

[13] Goldberg, M. C. et al. Developmental Cognitive Neuroscience Children with high functioning autism show increased prefrontal and temporal cortex activity during error monitoring. *Accid. Anal. Prev.* **1**, 47–56 (2011). 10.1016/j.dcn.2010.07.002 PMC2999812 21151713

[14] Sunkin, S. M. et al. Patches of Disorganization in the Neocortex of Children with Autism. 1209–1219 10.1056/NEJMoa1307491 (2014). 10.1056/NEJMoa1307491 PMC4499461 24670167

[15] Scoles, H. A., Urraca, N., Chadwick, S. W., Reiter, L. T. & Lasalle, J. M. Increased copy number for methylated maternal 15q duplications leads to changes in gene and protein expression in human cortical samples Increased copy number for methylated maternal 15q duplications leads to changes in gene and protein expression in human. **19**, (2011). 10.1186/2040-2392-2-19 PMC3287113 22152151

[16] Kadoshima, T. et al. Self-organization of axial polarity, inside-out layer pattern, and species-speci fi c progenitor dynamics in human ES cell – derived neocortex. 10.1073/pnas.1315710110 (2013). 10.1073/pnas.1315710110 PMC3864329 24277810

[17] Evolution, H. B. et al. Establishing Cerebral Organoids as Models of Article Establishing Cerebral Organoids as Models of Human-Specific Brain Evolution. *Cell* **176**, 743–756.e17 (2019). 30735633 10.1016/j.cell.2019.01.017 PMC6544371

[18] Hodge, R. D. et al. Conserved cell types with divergent features in human versus mouse cortex. *Nature* **573**, 61–68 (2019). 31435019 10.1038/s41586-019-1506-7 PMC6919571

[19] Caglayan, E., Liu, Y. & Konopka, G. Neuronal ambient RNA contamination causes misinterpreted and masked cell types in brain single-nuclei datasets. *Neuron* **110**, 4043–4056.e5 (2022). 36240767 10.1016/j.neuron.2022.09.010 PMC9789184

[20] Pollen, A. A. et al. Molecular identity of human outer radial glia during cortical development. *Cell* **163**, 55–67 (2015). 26406371 10.1016/j.cell.2015.09.004 PMC4583716

[21] Mouse and human share conserved transcriptional programs for interneuron development. **1342**, (2021). 10.1126/science.abj6641 PMC7618238 34882453

[22] Nowakowski, T. J. et al. Spatiotemporal gene expression trajectories reveal developmental hierarchies of the human cortex. **1323**, 1318–1323 (2017). 10.1126/science.aap8809 PMC5991609 29217575

[23] Lin, X., Chau, C., Ma, K., Huang, Y. & Ho, J. W. K. DCATS: differential composition analysis for flexible single-cell experimental designs. *Genome Biol.* **24**, 151 (2023). 37365636 10.1186/s13059-023-02980-3 PMC10294334

[24] Finak, G. et al. MAST: a flexible statistical framework for assessing transcriptional changes and characterizing heterogeneity in single-cell RNA sequencing data. *Genome Biol.* **16**, 278 (2015). 26653891 10.1186/s13059-015-0844-5 PMC4676162

[25] Dias, C. et al. Cell-type-specific effects of autism-associated 15q duplication syndrome in the human brain. *Am. J. Hum. Genet.* **111**, 1544–1558 (2024). 39079538 10.1016/j.ajhg.2024.07.002 PMC11339625

[26] Bhaduri, A. et al. Cell stress in cortical organoids impairs molecular subtype specification. *Nature* **578**, 142–148 (2020). 31996853 10.1038/s41586-020-1962-0 PMC7433012

[27] Zhang, Q. et al. hTFtarget: A Comprehensive Database for Regulations of Human Transcription Factors and Their Targets. *Genomics. Proteom. Bioinforma.* **18**, 120–128 (2020). 10.1016/j.gpb.2019.09.006 PMC7647694 32858223

[28] He, Z. et al. An integrated transcriptomic cell atlas of human neural organoids. *bioRxiv* 2023.10.05.561097 (2023).

[29] Iwata, R. & Vanderhaeghen, P. Regulatory roles of mitochondria and metabolism in neurogenesis. *Curr. Opin. Neurobiol.* **69**, 231–240 (2021). 34171617 10.1016/j.conb.2021.05.003 PMC8415079

[30] Zheng, X. et al. Metabolic reprogramming during neuronal differentiation from aerobic glycolysis to neuronal oxidative phosphorylation. *Elife.* **5**, e13374 (2016). 10.7554/eLife.13374 PMC4963198 27282387

[31] Ghosh-Choudhary, S., Liu, J. & Finkel, T. Metabolic Regulation of Cell Fate and Function. *Trends Cell Biol.* **30**, 201–212 (2020). 31983571 10.1016/j.tcb.2019.12.005 PMC7043867

[32] Traxler, L. et al. Warburg-like metabolic transformation underlies neuronal degeneration in sporadic Alzheimer’s disease. *Cell Metab.* **34**, 1248–1263.e6 (2022). 35987203 10.1016/j.cmet.2022.07.014 PMC9458870

[33] Mitelman, S. A. et al. Positron emission tomography assessment of cerebral glucose metabolic rates in autism spectrum disorder and schizophrenia. *Brain Imaging Behav.* **12**, 532–546 (2018). 28425060 10.1007/s11682-017-9721-z PMC5648637

[34] Frye, R. E. Mitochondrial Dysfunction in Autism Spectrum Disorder: Unique Abnormalities and Targeted Treatments. *Semin. Pediatr. Neurol.* **35**, 100829 (2020). 32892956 10.1016/j.spen.2020.100829

[35] Vallée, A. & Vallée, J.-N. Warburg effect hypothesis in autism Spectrum disorders. *Mol. Brain* **11**, 1 (2018). 29301575 10.1186/s13041-017-0343-6 PMC5753567

[36] Srour, M. et al. Dysfunction of the Cerebral Glucose Transporter SLC45A1 in Individuals with Intellectual Disability and Epilepsy. *Am. J. Hum. Genet.* **100**, 824–830 (2017). 28434495 10.1016/j.ajhg.2017.03.009 PMC5420346

[37] Abrahams, B. S. et al. SFARI Gene 2.0: a community-driven knowledgebase for the autism spectrum disorders (ASDs). *Mol. Autism* **4**, 36 (2013). 24090431 10.1186/2040-2392-4-36 PMC3851189

[38] Bjorklund, G. R. et al. Hyperactivation of MEK1 in cortical glutamatergic neurons results in projection axon deficits and aberrant motor learning. *DMM Dis. Model. Mech.* **17**, 1–18 (2024). 10.1242/dmm.050570 PMC11247507 38826084

[39] Pucilowska, J. et al. The 16p11.2 deletion mouse model of autism exhibits altered cortical progenitor proliferation and brain cytoarchitecture linked to the ERK MAPK pathway. *J. Neurosci.* **35**, 3190–3200 (2015). 25698753 10.1523/JNEUROSCI.4864-13.2015 PMC6605601

[40] O’Roak, B. J. et al. Multiplex targeted sequencing identifies recurrently mutated genes in autism spectrum disorders. *Science* **338**, 1619–1622 (2012). 23160955 10.1126/science.1227764 PMC3528801

[41] Gao, Z. et al. An AUTS2–Polycomb complex activates gene expression in the CNS. *Nature* **516**, 349–354 (2014). 25519132 10.1038/nature13921 PMC4323097

[42] Pino, E. et al. FOXO3 determines the accumulation of α-synuclein and controls the fate of dopaminergic neurons in the substantia nigra. *Hum. Mol. Genet.* **23**, 1435–1452 (2014). 24158851 10.1093/hmg/ddt530

[43] Schäffner, I. et al. FoxO Function Is Essential for Maintenance of Autophagic Flux and Neuronal Morphogenesis in Adult Neurogenesis. *Neuron* **99**, 1188–1203.e6 (2018). 30197237 10.1016/j.neuron.2018.08.017 PMC6186958

[44] Wegiel, J. et al. Significant neuronal soma volume deficit in the limbic system in subjects with 15q11.2-q13 duplications. *Acta Neuropathol. Commun.* **3**, 63 (2015). 26463344 10.1186/s40478-015-0241-z PMC4603300

[45] Stuart, T. et al. Comprehensive Integration of Single-Cell Data. *Cell* **177**, 1888–1902.e21 (2019). 31178118 10.1016/j.cell.2019.05.031 PMC6687398

[46] Butler, A., Hoffman, P., Smibert, P., Papalexi, E. & Satija, R. Integrating single-cell transcriptomic data across different conditions, technologies, and species. *Nat. Biotechnol.* **36**, 411–420 (2018). 29608179 10.1038/nbt.4096 PMC6700744

[47] Caglayan, E. et al. Ambient RNA analysis reveals misinterpreted and masked cell types in brain single-nuclei datasets. (2022). 10.1016/j.neuron.2022.09.010 PMC9789184 36240767

[48] Velmeshev, D. et al. Single-cell genomics identifies cell type-specific molecular changes in autism. *Science* **364**, 685–689 (2019). 31097668 10.1126/science.aav8130 PMC7678724

[49] Cao, J. et al. The single-cell transcriptional landscape of mammalian organogenesis. *Nature* **566**, 496–502 (2019). 30787437 10.1038/s41586-019-0969-x PMC6434952

[50] Liberzon, A. et al. The Molecular Signatures Database Hallmark Gene Set Collection. *Cell Syst.* **1**, 417–425 (2015). 26771021 10.1016/j.cels.2015.12.004 PMC4707969

[51] Morabito, S., Reese, F., Rahimzadeh, N., Miyoshi, E. & Swarup, V. High dimensional co-expression networks enable discovery of transcriptomic drivers in complex biological systems. 1–32 (2022).

[52] Kuleshov, M. V. et al. Enrichr: a comprehensive gene set enrichment analysis web server 2016 update. **44**, 90–97 (2016). 10.1093/nar/gkw377 PMC4987924 27141961

[53] Mi, H., Muruganujan, A., Casagrande, J. T. & Thomas, P. D. Large-scale gene function analysis with the PANTHER classification system. *Nat. Protoc.* **8**, 1551–1566 (2013). 23868073 10.1038/nprot.2013.092 PMC6519453

[54] Bardy, C. et al. Neuronal medium that supports basic synaptic functions and activity of human neurons in vitro. *Proc. Natl Acad. Sci.* **112**, E2725–E2734 (2015). 25870293 10.1073/pnas.1504393112 PMC4443325

[55] Paredes, M. F. et al. Nests of dividing neuroblasts sustain interneuron production for the developing human brain. *Science* **375**, eabk2346 (2022). 35084970 10.1126/science.abk2346 PMC8887556

