# Supplementary material for: Single-cell analysis of dup15q syndrome reveals developmental and postnatal molecular changes in autism
Source: Nat Commun. 2025 Jul 4;16:6177. doi: 10.1038/s41467-025-61184-4 (PMC12227528; doi:10.1038/s41467-025-61184-4)
Supplement: Supplementary file 1 — Supplementary Information [file 41467_2025_61184_MOESM1_ESM.docx]

Supplementary Figures:

Title: Single-cell analysis of dup15q syndrome reveals developmental and postnatal molecular changes in autism.

**Authors:** Yonatan Perez^1,2^*†, Dmitry Velmeshev^1,2,3^*, Li Wang^1,2^, Matthew L White^1,2^, Clara Siebert^1,2^, Jennifer Baltazar^1,2^, Guolong Zuo^1,2^, Juan Andrés Moriano^1,2^, Songcang Chen^1,2^, David M Steffen^1^, Natalia Garcia Dutton^1,2^, Shaohui Wang^1,2^, Brittney Wick^4^, Maximilian Haeussler^4^, Stormy Chamberlain^5^, Arturo Alvarez-Buylla^1^ and Arnold Kriegstein^1,2^†

†Corresponding authors: arnold.kriegstein@ucsf.edu and jonatan.perez@ucsf.edu.

*These authors contributed equally to this work.

**Supplementary Figures**

**
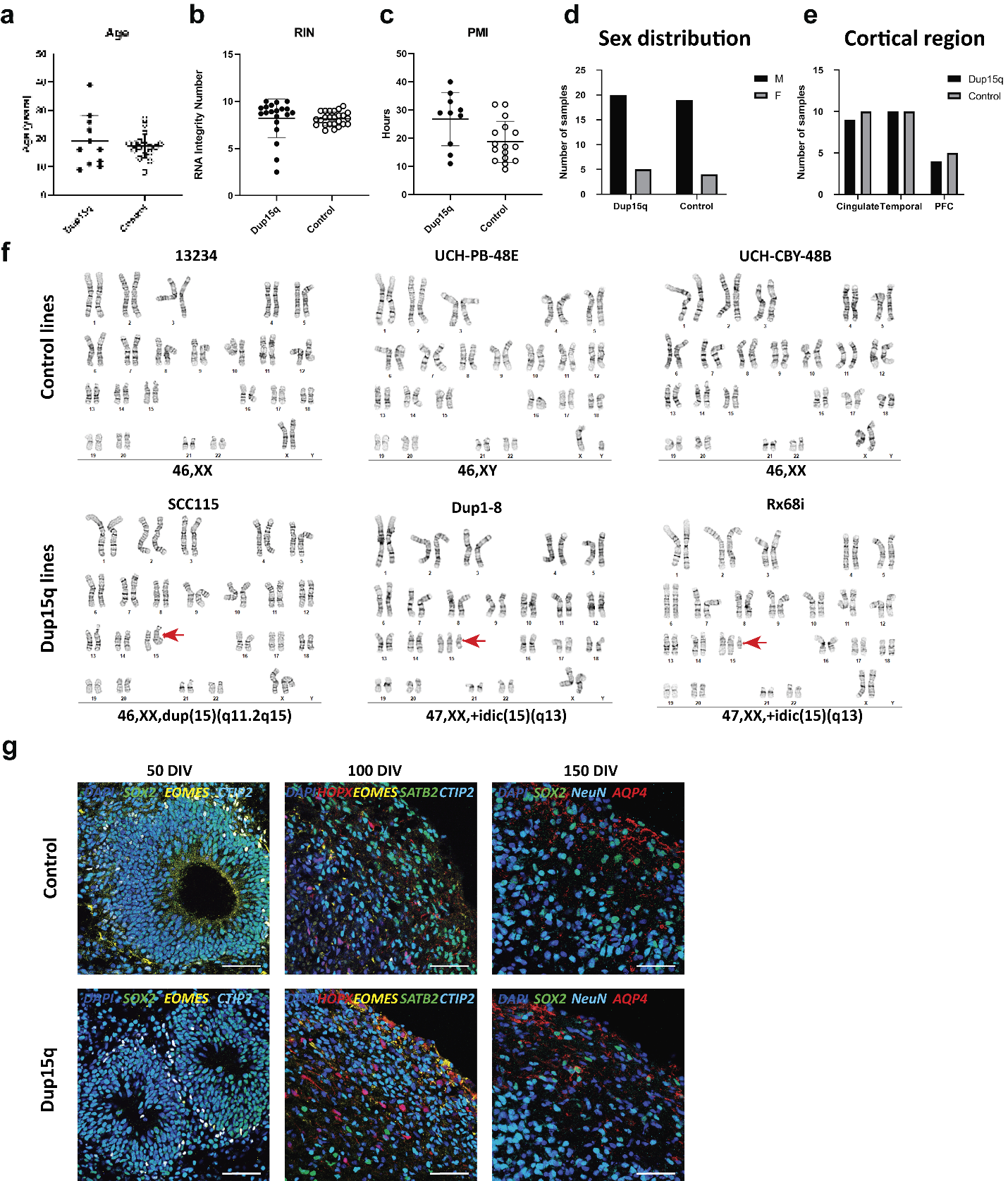
**

**Supplementary Fig. S1. Sample statistics for experimental groups, iPSC karyotype and organoid development. a-e)** Comparison of age, RNA integrity number (RIN), post-mortem interval (PMI), sex, and brain regions between control and dup15q groups. **f)** G-banding karyotype analysis of all iPSC lines used in the study**. g)** Immunostainings of organoid sections at three sampling developmental timepoints (50, 100 and 150 days of *in-vitro* development, scale bar = 50𝜇m).


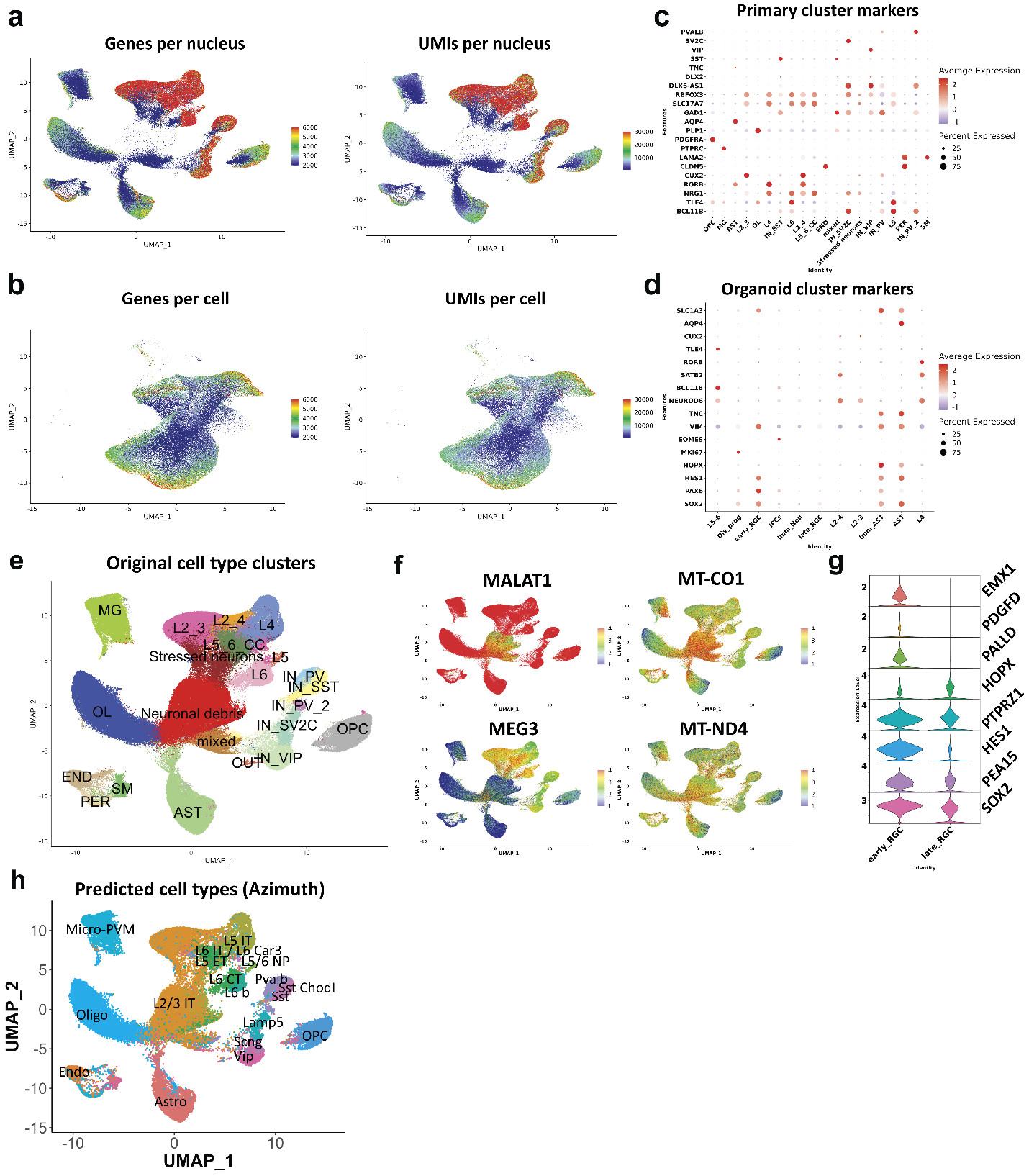


**Supplementary Fig. S2. Technical and biological characteristics of the primary and organoid datasets. a)** Primary gene and UMI counts per nucleus across all cell types. **b)** Organoid gene and UMI counts per cell across all cell types. **c)** Primary nuclei marker gene expression used to annotate specific cells. **d)** Organoid marker gene expression used to annotate specific cells. **e-f)** Identification of clusters containing neuronal debris, expression low nuclear-retention and high mitochondrial transcripts. **g)** Marker gene expression of early radial glia (early_RGC) vs late radial glia cells (late_RSC). **h)** Predicted cell types when reference-based mapping to the Azimuth human motor cortex dataset.

**
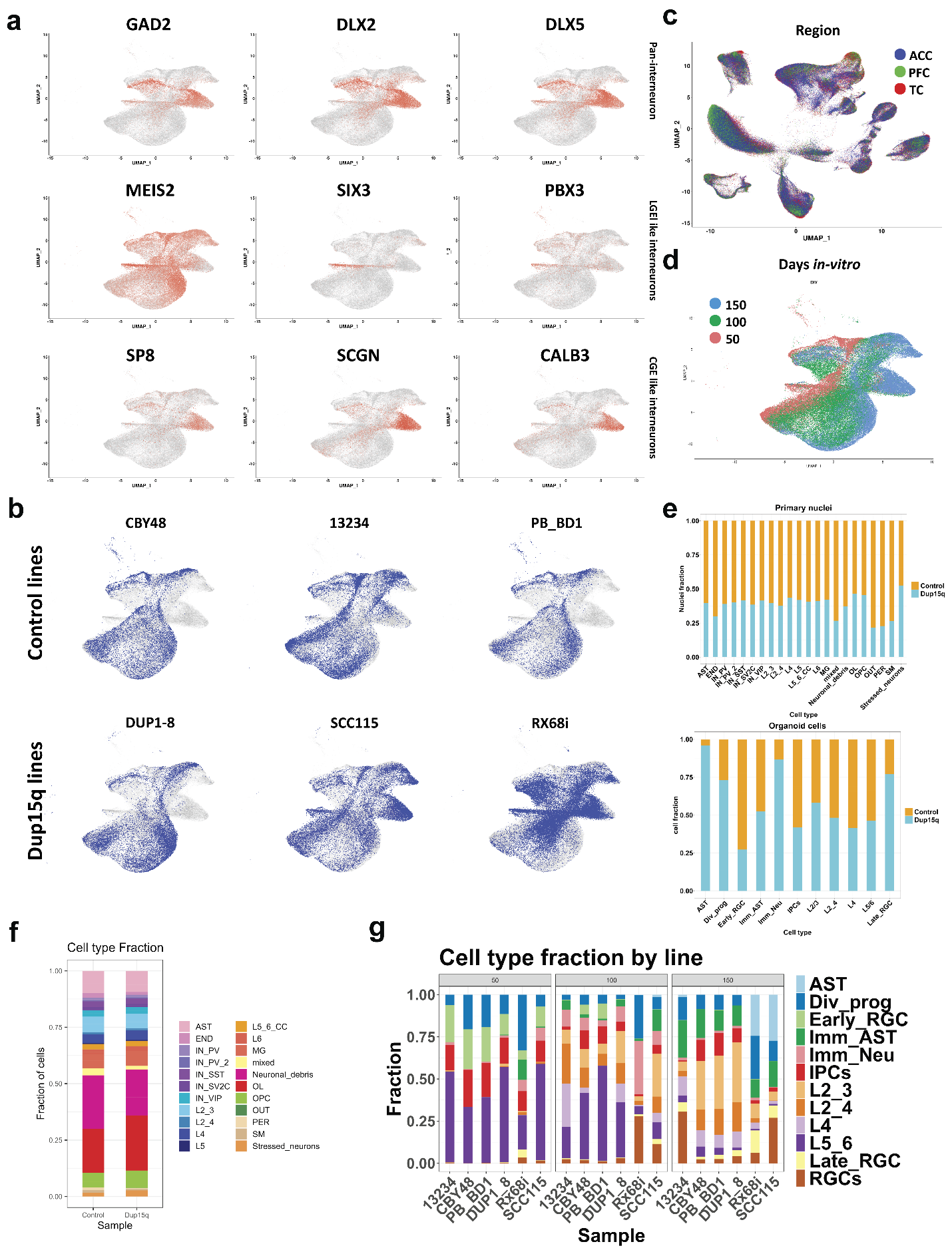
**

**Supplementary Fig. S3. Organoid interneuron identification and cell-type proportions. a)** Organoid marker gene expression identifies interneuron subtypes. **b)** Contribution of each line to specific organoid cell types (cells from a specific line are in blue; gray are cells from all other lines). **c)** UMAP of all primary cell types grouped by cortical region. **d)** UMAP of organoid cell-types grouped by differentiation timepoint. **e)** Stacked histogram showing cell type proportions and cell type fraction by cell-type for dup15q and control primary nuclei. **f)** Stacked histogram showing cell type proportions and cell type fraction by cell-type for dup15q and control organoid cells. **g)** Proportion of each cell type per line, split by days of in vitro development.

**
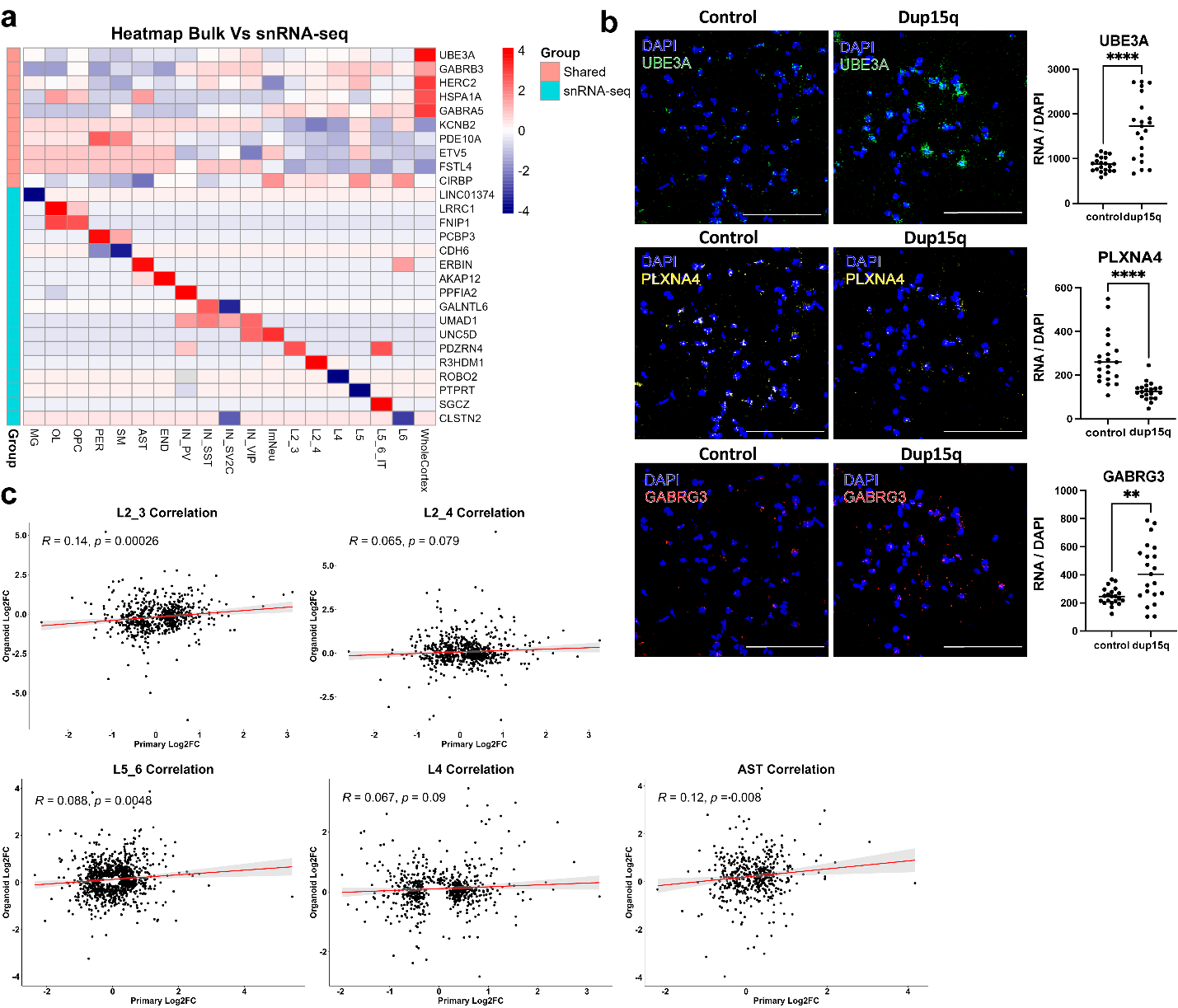
**

**Supplementary Fig. S4. Single-molecule RNA *in situ* hybridization validation of selected differentially expressed genes across all cell types of dup15q and control cortical samples. a)** Heatmap showing examples of ubiquitously expressed genes identified by a previous bulk RNA analysis (WholeCortex) and by our snRNA analysis (Shared in Salmon) vs cell-type specific genes changes identified only in this study (snRNA-seq in cyan). **b)** Validation of *UBE3A, PLXNA4,* and *GABRG3* transcripts in neurons of the temporal cortex. (n=3 cortical samples from both dup15q and controls. assay was performed on sections from temporal cortex (BA20). Statistics represent counts of 7 images each. Scale bar = 100µm). **c)** Correlation coefficient scatterplots for all genes significant in either primary or organoids cells (R represents Pearson’s correlation coefficient, p represents Pearson’s p value).

**
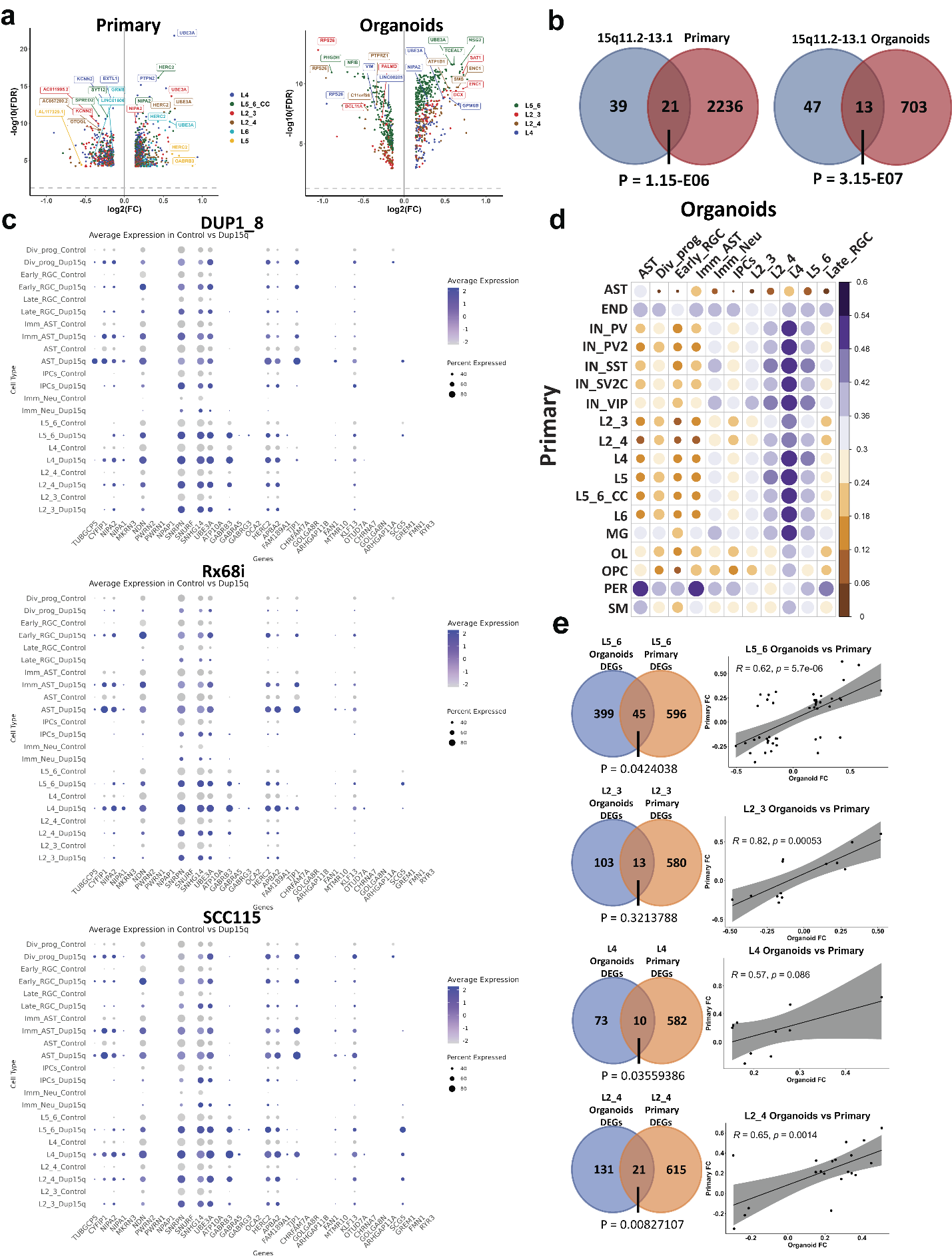
**

**Supplementary Fig. S5. Contribution of duplicated genes to DEGs and cell type-specific gene expression correlation. a)** Volcano plots for neuron-specific genes most differentially expressed in both primary and organoid excitatory neurons. **b)** Venn diagram showing the overlap between putative duplicated genes with all primary and organoid overexpressed genes pooled from all cell types (P denotes a hypergeometric p-value). **c)** Average expression of genes (Wilcoxon rank-sum test) within the duplicated chromosome 15 locus for each organoid line separately. **d)** Pearson’s correlation plot of the top 100 expressed genes between organoid and primary cell types. **e)** On the left are Venn diagrams showing overlapping DEGs (P values of intersection represent Fisher’s exact test P values). On the right is the correlation coefficient of intersected gene fold changes between comparable primary and organoid neurons.


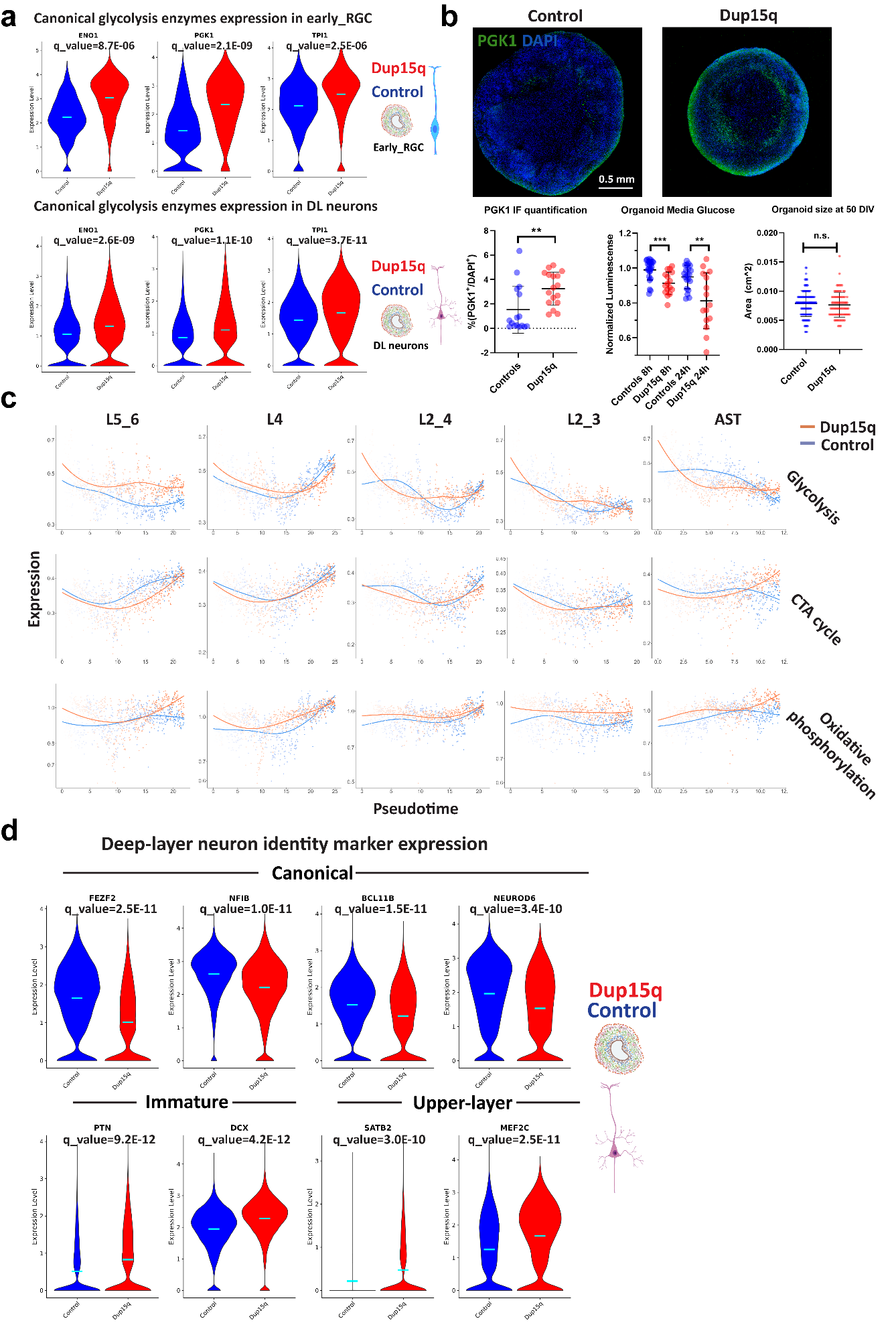


**Supplementary Fig. S6. Enriched Glycolysis leads to degraded marker identity in organoid DL neurons. a)** Violin plots showing increased expression of canonical glycolytic enzymes in dup15q early RGCs and DL neurons (q_values represent FDR-corrected for multiple comparisons). **b)** Immunofluorescent staining of the glycolysis enzyme PGK1 in control and dup15q organoids. The plots below show a quantification summary of PGK1 expression, organoid media glucose levels as a proxy for organoid glucose intake, and organoid size at 50DIV (p values were calculated using an unpaired t-test). **c)** Metabolic gene set expression levels of all organoid cell-specific trajectories plotted along pseudotime (red line denotes dup15q while blue lines denote control expression). **d)** Violin plots showing co-expression of DL markers, immature neuron markers, and UL neuron markers, indicating degraded identity of dup15q organoid DL neurons. (Control in blue and dup15q in red, q_values represent FDR-corrected for multiple comparisons).


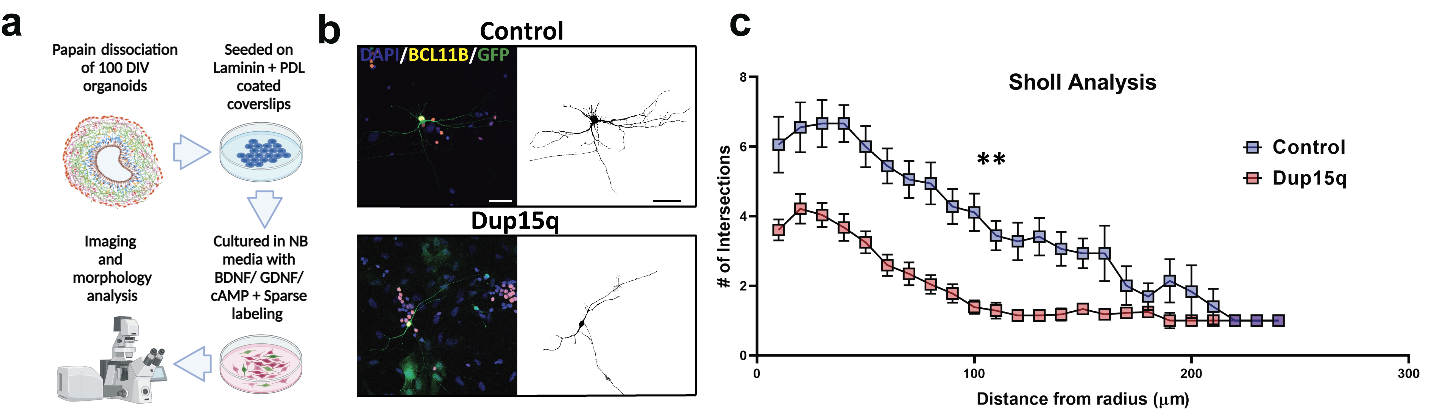


**Supplementary Fig. S7. Dup15q deep-layer neurons exhibit aberrant morphology in vitro.**

**a)** Illustration of cortical organoid cell dissociation, maturation, sparse labeling and morphological analysis in 2D. Some elements were created in BioRender. Perez, J. (2025) <https://BioRender.com/8ruoefr>. **b)** IF-staining and deep-layer neurite tracing for Sholl analysis (Scale bar = 50𝜇m). **c)** Sholl analysis of deep-layer neurons. (unpaired t-test with a p value < 0.005 was achieved for all measurements between 10-150𝜇m distance from radius; n=18 for controls; n=28 for dup15q).

**
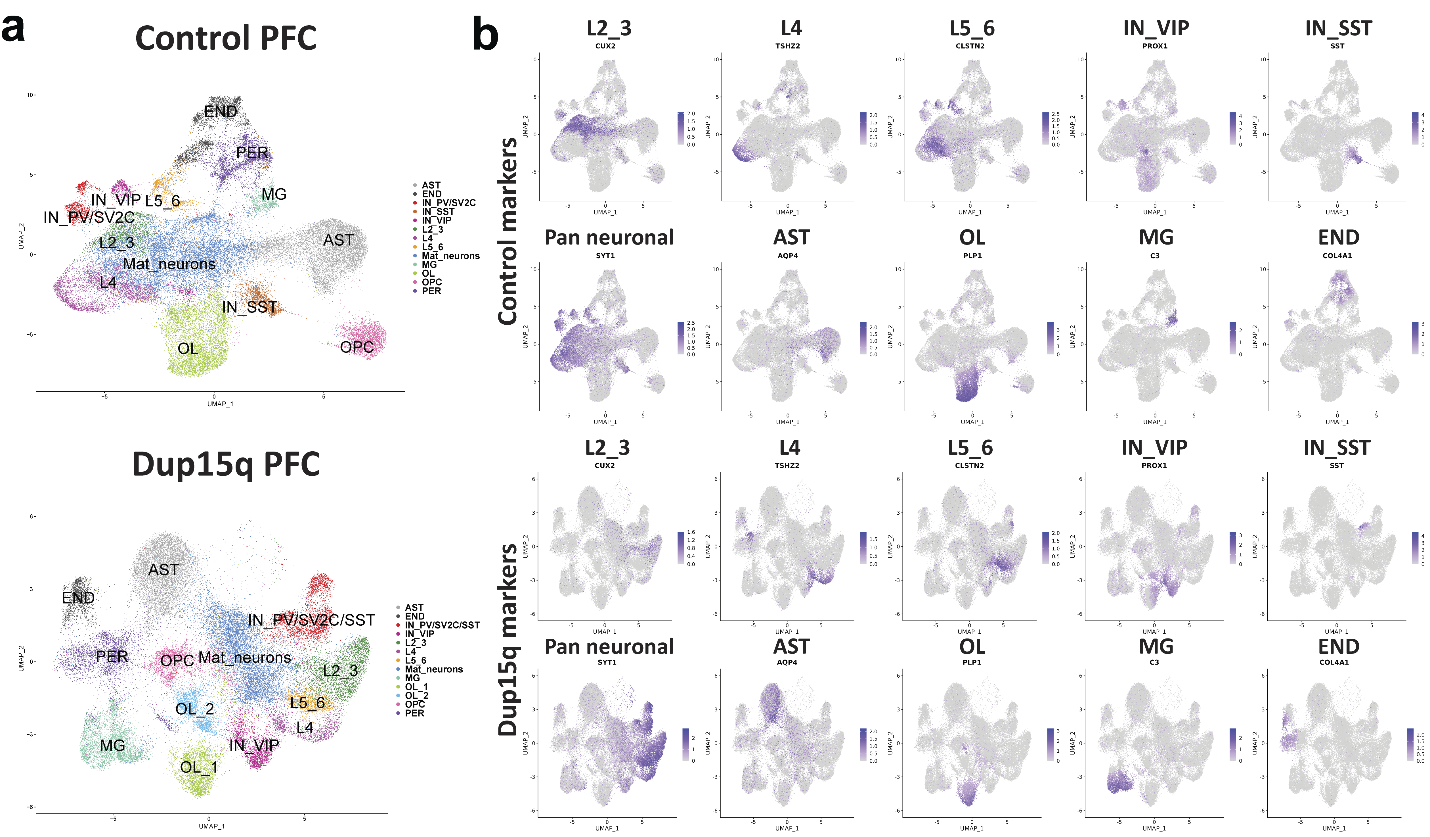
**

**Supplementary Fig. S8. Spatial transcriptomics clustering and annotation. a)** Uniform Manifold Approximation and Projection (UMAP) embedding and cluster annotations of single cell spatial transcriptomic data. **b)** Cell subtype gene expression of markers used for cluster annotations.


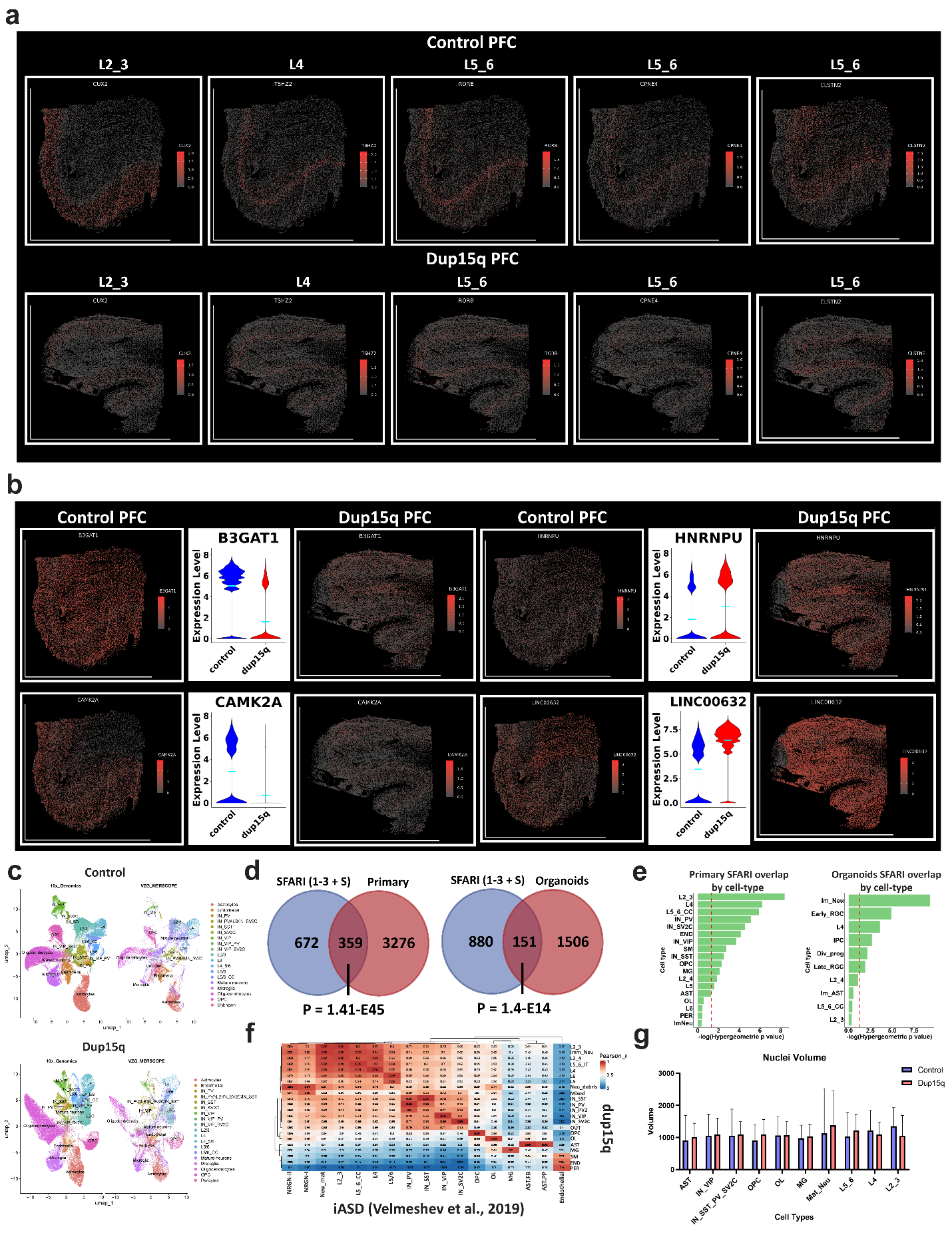


**Supplementary Fig. S9. Spatial transcriptomics validates differential gene expression in dup15q syndrome PFC. a)** Excitatory neuron layer-specific markers tissue expression. **b)** Examples of differentially expressed genes, validated through spatial resolved transcriptomics. **c)** snRNA data and MERSCOPE data integration based on canonical correlation analysis. **d)** Venn diagram showing the overlap between primary and organoid DEGs and high-confidence ASD genetic risk factors (SFARI gene scores 1 to 3 and syndromic; Hypergeometric P-value). **e)** Overlap between SFARI genes and DEGs by cell-type; dashed red line indicates statistical significance (q < 0.05). **f)** Heatmap with Pearson’s correlation plot of highly expressed genes between dup15q and idiopathic ASD cell types. **g)** Mean nuclear volume, measured via spatial transcriptomics, as proxy for soma size. Data are presented as mean values +/- SD.


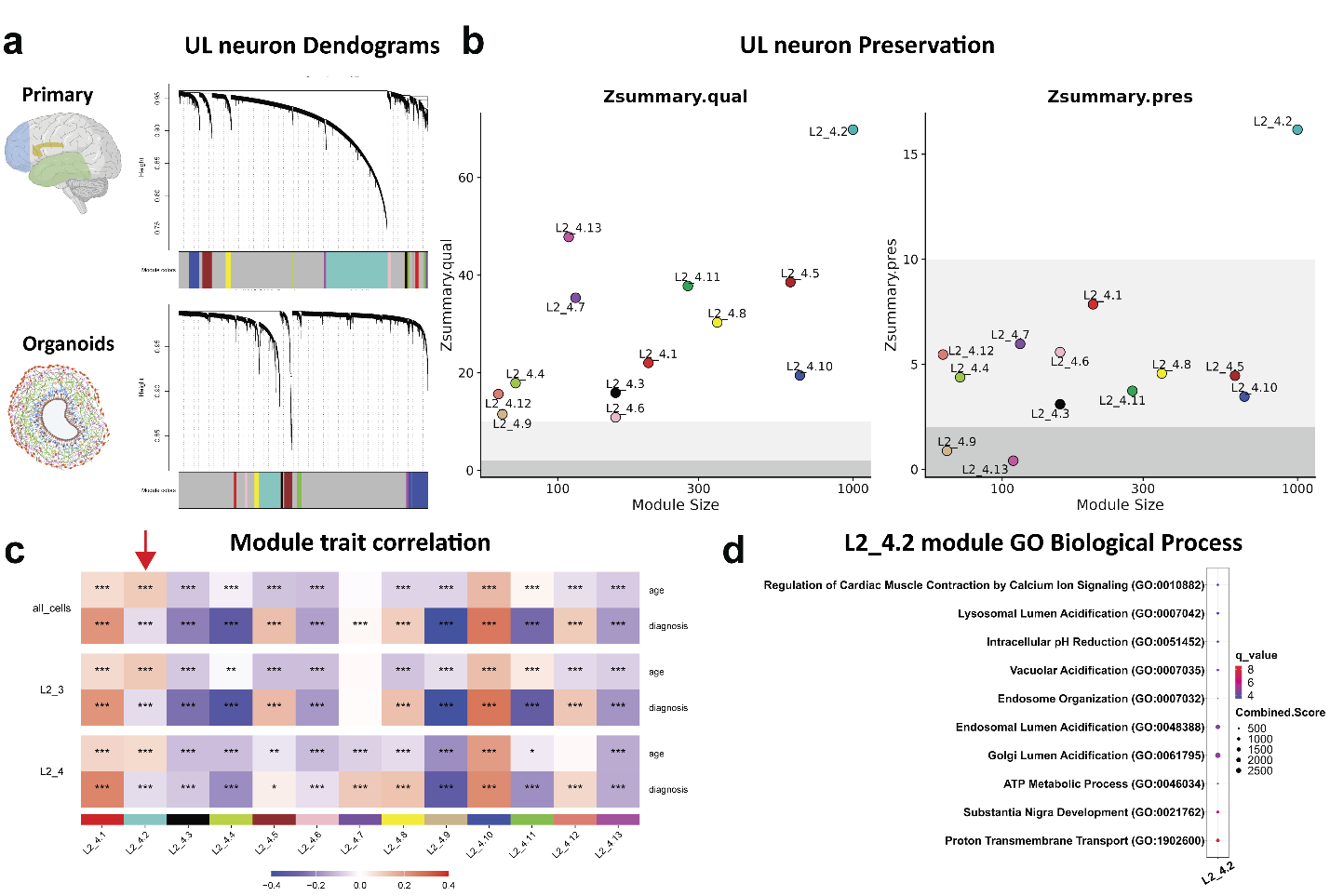


**Supplementary Fig. S10. Weighted gene co-expression networks (WGCNA) of organoid and primary dup15q UL neurons. a)** WGCNA dendrograms of primary and organoid upper-layer (UL) neurons. Each leaf represents a single gene, and the colors on the bottom represent the assignment of co-expression modules. **b)** Module preservation analysis of primary UL neurons. (Zsummary.qual scores >10 indicate high robustness of module quality; Zsummary.pres >10 indicates strong preservation of the primary modules in the organoid network). **c)** Module trait correlation analysis of primary UL neuron modules. The red arrows indicate the downregulated L2_4.2 well-preserved module associated with the dup15q genotype. d) Gene ontology analysis showing enriched biological processes of the L2_4.2 module.
