# Supplementary material for: Single-cell analysis of dup15q syndrome reveals developmental and postnatal molecular changes in autism
Source: Nat Commun. 2025 Jul 4;16:6177. doi: 10.1038/s41467-025-61184-4 (PMC12227528; doi:10.1038/s41467-025-61184-4)
Supplement: Supplementary file 2 — Description of Additional Supplementary Files [file 41467_2025_61184_MOESM2_ESM.pdf]

Description of Additional Supplementary Files

Supporting Information

Source Data (provided as a separate .xlsx file)
Supplementary Figures (provided as a separate .docx file)
Supplementary Datasets (each dataset is provided as a separate .xlsx file)

File Name: Supplementary Dataset S1. Sample metadata.
Description: This table summarizes sample-level metadata for all donors included in the transcriptomic analyses.

File Name: Supplementary Dataset S2. Relative cell proportion analysis.
Description: Proportional changes in cell-type abundance were estimated using the DCATS method. Coefficients represent log fold changes in cell-type abundance between Dup15q and control samples. (coeffs: Estimated log fold change; coeffs_err; Standard error; LRT_pvals: Likelihood ratio test p-values; fdr: FDR-adjusted p-values using the Benjamini–Hochberg method; ratio: Ratio of Dup15q to control cell proportions.

File Name: Supplementary Dataset S3. Nuclei-specific differentially expressed genes of dup15q cortex.
Description: Differentially expressed genes (DEGs) between Dup15q and control primary samples were identified for each cell type using MAST (model was fit for primary nuclei as in Methods). P-values are from likelihood ratio tests comparing models with and without the diagnosis term. All tests were two-sided and corrected for multiple comparisons using the Benjamini–Hochberg FDR method.

File Name: Supplementary Dataset S4. Cell-type specific differentially expressed genes of dup15q organoids.
Description: Differentially expressed genes (DEGs) between Dup15q and control organoid samples were identified for each cell type using MAST (model was fit for Organoid cells as in Methods). P-values are from likelihood ratio tests comparing models with and without the diagnosis term. All tests were two-sided and corrected for multiple comparisons using the Benjamini–Hochberg FDR method.

File Name: Supplementary Dataset S5. Dup15q bulk comparison.
Description: Pearson's correlation and hypergeometric tests were used to evaluate consistency between datasets. (Pearson's r: Correlation of log2 fold changes; p value: Two-sided p-value for Pearson's r; hypergeometric p: Two-sided hypergeometric test for DEG overlap; adjusted p value: FDR-corrected hypergeometric p-value (Benjamini–Hochberg method); All statistical tests were two-sided.

File Name: Supplementary Dataset S6. Transcription Factors (TFs) target prediction.

Description: Differentially expressed TFs identified in organoid cells were analyzed using hTFtarget to predict target genes. Cell type–specific overlaps with DEGs from primary brain cells were assessed using a one-sided hypergeometric test.

File Name: Supplementary Dataset S7. Permutation analysis.
Description: To assess genotype-specific effects, we performed permutation testing by shuffling sample IDs and recalculating pathway-level statistics for each permutation. Empirical p-values and FDR were derived from the null distribution generated across 10 iterations.

File Name: Supplementary Dataset S8. Lineage-specific differentially expressed genes of dup15q organoids.
Description: To identify genes dynamically dysregulated in Dup15q cortical organoids, we compared lineage-specific pseudotime trajectories between control and Dup15q samples. Differential expression scores were calculated as the difference in the area under the smoothed gene expression curves over pseudotime between conditions. Genes with a differential expression score ≥50 were considered differentially expressed. For L5_6 lineages, differential expression was further tested using tradeSeq (fitGAM and patternTest), and p-values were combined using the logit method (allmetap, metap package). Reported p-values were adjusted for multiple testing using the Benjamini–Hochberg method.

File Name: Supplementary Dataset S9. Genes used for spatial transcriptomics.
Description: The codebook used for MERSCOPE experiments.

File Name: Supplementary Dataset S10. Cortical region-specific differentially expressed genes.
Description: Differential expression analysis was performed separately for each cortical region (frontal cortex [FC], cingulate cortex [CC], and temporal cortex [TC]) using MAST with a generalized linear mixed model (GLMM). The model included donor as a random effect and adjusted for relevant covariates (age, sex, cngeneson, RIN, PMI, Capbatch, Seqbatch, ribo_perc). Region-specific comparisons tested for expression differences between Dup15q and control samples within each region. All tests were two-sided, and FDR correction was applied using the Benjamini–Hochberg method. Genes with FDR < 0.05 were considered significantly differentially expressed.

File Name: Supplementary Dataset S11. Weighted gene co-expression modules of primary and organoid DL neurons.
Description: Genes were assigned to co-expression modules using hdWGCNA analysis of deep-layer (DL) neurons from primary brain tissue and cortical organoids. Correlation between gene expression and the module eigengene (kME) is reported across modules.

File Name: Supplementary Dataset S12. Weighted gene co-expression modules of primary and organoid UL neurons.
Description: Genes were assigned to co-expression modules using hdWGCNA analysis of upper-layer (UL) neurons from primary brain tissue and cortical organoids. Correlation between gene expression and the module eigengene (kME) is reported across modules.
