# Supplementary material for: Single-cell analysis of dup15q syndrome reveals developmental and postnatal molecular changes in autism
Source: Nat Commun. 2025 Jul 4;16:6177. doi: 10.1038/s41467-025-61184-4 (PMC12227528; doi:10.1038/s41467-025-61184-4)
Supplement: Supplementary file 15 — Reporting Summary [file 41467_2025_61184_MOESM15_ESM.pdf]

Corresponding author(s): Arnold Kriegatein
Yonatan Perez

Last updated by author(s): May 13, 2025

Reporting Summary

Nature Portfolio wishes to improve the reproducibility of the work that we publish. This form provides structure for consistency and transparency in reporting. For further information on Nature Portfolio policies, see our Editorial Policies and the Editorial Policy Checklist.

Statistics

For all statistical analyses, confirm that the following items are present in the figure legend, table legend, main text, or Methods section.

n/a	Confirmed	
☐	☒	The exact sample size (*n*) for each experimental group/condition, given as a discrete number and unit of measurement
☐	☒	A statement on whether measurements were taken from distinct samples or whether the same sample was measured repeatedly
☐	☒	The statistical test(s) used AND whether they are one- or two-sided<br>*Only common tests should be described solely by name; describe more complex techniques in the Methods section.*
☐	☒	A description of all covariates tested
☐	☒	A description of any assumptions or corrections, such as tests of normality and adjustment for multiple comparisons
☐	☒	A full description of the statistical parameters including central tendency (e.g. means) or other basic estimates (e.g. regression coefficient) AND variation (e.g. standard deviation) or associated estimates of uncertainty (e.g. confidence intervals)
☐	☒	For null hypothesis testing, the test statistic (e.g. *F*, *t*, *r*) with confidence intervals, effect sizes, degrees of freedom and *P* value noted<br>*Give P values as exact values whenever suitable.*
☐	☒	For Bayesian analysis, information on the choice of priors and Markov chain Monte Carlo settings
☐	☒	For hierarchical and complex designs, identification of the appropriate level for tests and full reporting of outcomes
☐	☒	Estimates of effect sizes (e.g. Cohen's *d*, Pearson's *r*), indicating how they were calculated

Our web collection on statistics for biologists contains articles on many of the points above.

Software and code

Policy information about availability of computer code

Data collection	All single cell/ single nucleus RNAseq data was collected form the Illumina Novaseq 6000 platform. Raw Binary Base Call (BCL) files were used for downstream analysis as described below.
Data analysis	CellRanger software v 6.1.1 was used for library demultiplexing, fastq file generation, read alignment and UMI quantification.<br>Seurat v.4. R package was used for QC, unbiased clustering and dimensionality reduction projection.<br>MAST r package was used to calculate differential gene expression and to perform zero-inflated regression analysis by fitting a linear mixed model (LMM).<br>Monocle3 R package was used for trajectory reconstruction. Moran's test (graph_test function) was used to identify genes that are dynamically expressed in each lineage.<br>The hdWGCNA R package was used to generate weighted gene co-expression networks.<br>The corrplot R package was used to calculate gene expression correlation.<br>The PANTHER v17.0 was used to perform statistical overrepresentation test for DEGs from each cluster.<br>Graphpad prism 8.0 software was used for RNA scope and immunohistochemistry.

For manuscripts utilizing custom algorithms or software that are central to the research but not yet described in published literature, software must be made available to editors and reviewers. We strongly encourage code deposition in a community repository (e.g. GitHub). See the Nature Portfolio guidelines for submitting code & software for further information.

Data

Policy information about availability of data

All manuscripts must include a data availability statement. This statement should provide the following information, where applicable:

- Accession codes, unique identifiers, or web links for publicly available datasets
- A description of any restrictions on data availability
- For clinical datasets or third party data, please ensure that the statement adheres to our policy

Raw data can be accessed at the Sequence Read Archive (SRA), accession number PRJNA1017130. Analyzed data (cell-count matrix and metadata) can be accessed through the UCSC Cell Browser, collection dup15q-cortexl-organoids (https://cells.ucsc.edu/?ds=dup15q-cortex-organoids).

Research involving human participants, their data, or biological material

Policy information about studies with human participants or human data. See also policy information about sex, gender (identity/presentation), and sexual orientation and race, ethnicity and racism.

Reporting on sex and gender	Sex was considered in the study design.<br>For postmortem samples, the sex was determined and supplied by the brain banks from which the tissue was provided (as described in Supplementary Table 1. Sample metadata).<br>For cell line used, sex was determined by Karyotype analysis.
Reporting on race, ethnicity, or other socially relevant groupings	Not specified in this study.
Population characteristics	Population characteristics as provided in The Extended Data Table 1. Sample metadata: individual ID, Brain region, Diagnosis, sex, age, postmortem interval (PMI), Brain_bank, Brain_bank_location, RNA integrity (RIN), diagnosis co-morbidities, psych_condition, ADI_Social, ADI_Ver_Comm, ADI_RRS, ADI_R, and karyotype.
Recruitment	All postmortem samples used in this study were provided by the Autism BrainNet and the NIH NeuroBioBank.
Ethics oversight	All samples were obtained and processed as approved by the UCSF Human Gamete, Embryo and Stem Cell Research Committee (GESCR, approval 10-05113). All experiments were performed in accordance with protocol guidelines.

Note that full information on the approval of the study protocol must also be provided in the manuscript.

Field-specific reporting

Please select the one below that is the best fit for your research. If you are not sure, read the appropriate sections before making your selection.

☒ Life sciences ☐ Behavioural & social sciences ☐ Ecological, evolutionary & environmental sciences

For a reference copy of the document with all sections, see nature.com/documents/nr-reporting-summary-flat.pdf

Life sciences study design

All studies must disclose on these points even when the disclosure is negative.

Sample size	No statistical methods were used to predetermine sample size
Data exclusions	No exclusions were made.
Replication	It is not feasible to replicate findings in this study. However, IF stainings, RNA in situ hybridization, and spatial transcriptomic analyses were used to validate findings.
Randomization	Both dup15q and control postmortem samples were matched for age, sex, RNA integrity number (RIN), and post-mortem interval (PMI).
Blinding	The investigator was blinded to sample labels before acquiring and analyzing the RNA in-situ hybridization images.<br>Investigators were not blinded to allocation during other experiments and outcome assessments.

Reporting for specific materials, systems and methods

We require information from authors about some types of materials, experimental systems and methods used in many studies. Here, indicate whether each material, system or method listed is relevant to your study. If you are not sure if a list item applies to your research, read the appropriate section before selecting a response.

Materials & experimental systems

n/a	Involved in the study
☐	☒ Antibodies
☐	☒ Eukaryotic cell lines
☒	☐ Palaeontology and archaeology
☐	☒ Animals and other organisms
☐	☒ Clinical data
☒	☐ Dual use research of concern
☒	☐ Plants

Methods

n/a	Involved in the study
☒	☐ ChIP-seq
☒	☐ Flow cytometry
☒	☐ MRI-based neuroimaging

Antibodies

Antibodies used

MBP, goat, Santa cruz bio, SC-13914, 1:500
SOX2, Mouse, Santa cruz bio, sc-365823, 1:500
EOMES, Sheep, R&D systems, AF6166, 1:500
BCL11B, Rat, Abcam, ab18465, 1:500
HOPX, Rabbit, Proteintech, 11419-1-AP, 1:500
SATB2, mouse, Abcam, ab51502, 1:500
NeuN, Guinea pig, Millipore, ABN90, 1:500
AQP4, Rabbit, Proteintech, 16473-1-AP, 1:500
PGK1, Rabbit, ThermoFisher, PA5-13863, 1:100

Validation

All antibodies are routinely used in our lab and have been validated on multiple tissue types. Further validation can be found in manufacturer's website.

Eukaryotic cell lines

Policy information about cell lines and Sex and Gender in Research

Cell line source(s)

1. Line: SCC115-6. Source: Fibroblasts. Reprograming: Sendai. Sex: Female. Diagnosis: dup15q syndrome. Karyotype: Mat.int.trip. line source: Uconn stem cell core.
2. Line: DUP1_8. Source: Fibroblasts. Reprograming: Retrovirus (Y4 + Lin28). Sex: Male. Diagnosis: dup15q syndrome. Karyotype: idic(15). line source: Uconn stem cell core.
3. Line: Rx68i. Source: Cord blood. Reprograming: Episomal (Y4). Sex: female. Diagnosis: dup15q syndrome. Karyotype: idic(15). line source: Uconn stem cell core.
4. Line: PB_BD1. Source: Peripheral blood. Reprograming: Episomal/ OCT4, SOX2, KLF4, L-MYC, LIN28, and P53-shRNA. Sex: Male. Diagnosis: Control. Karyotype: Normal. line source: Uconn stem cell core.
5. Line: CBY48. Source: Cord blood. Reprograming: Episomal/ OCT4, SOX2, KLF4, L-MYC, LIN28, and P53-shRNA. Sex: Female. Diagnosis: Control. Karyotype: Normal. line source: Uconn stem cell core.
6. Line: 13234. Source: Fibroblasts. Reprograming: Episomal (Okita et al., 2011). Sex: Female. Diagnosis: Control. Karyotype: Normal. line source: Conklin lab (Gladstone)

Authentication

All lines were authenticated by source laboratories. Pluripotency and karyotype anlyses were done in our lab.

Mycoplasma contamination

All cell lines were tested negative for mycoplasma

Commonly misidentified lines
(See ICLAC register)

Name any commonly misidentified cell lines used in the study and provide a rationale for their use.

Animals and other research organisms

Policy information about studies involving animals; ARRIVE guidelines recommended for reporting animal research, and Sex and Gender in Research

Laboratory animals

For human xenotransplantation, we used both female and male NSG (NOD.Cg-Prkdcscid Il2rgtm1Wjl/SzJ) neonates at P4.

Wild animals

NA

Reporting on sex

NA

Field-collected samples | NA

Ethics oversight | Animal experiments were performed in accordance to the UCSF – Institutional Animal Care & Use Committee (protocol AN1883074-01D).

Note that full information on the approval of the study protocol must also be provided in the manuscript.

Clinical data

Policy information about clinical studies

All manuscripts should comply with the ICMJE guidelines for publication of clinical research and a completed CONSORT checklist must be included with all submissions.

Clinical trial registration | *Provide the trial registration number from ClinicalTrials.gov or an equivalent agency.*

Study protocol | *Note where the full trial protocol can be accessed OR if not available, explain why.*

Data collection | *Describe the settings and locales of data collection, noting the time periods of recruitment and data collection.*

Outcomes | *Describe how you pre-defined primary and secondary outcome measures and how you assessed these measures.*
